# Photoelectrocatalytic H_2_ evolution in water with molecular catalysts immobilised on p-Si *via* a stabilising mesoporous TiO_2_ interlayer†
†Electronic supplementary information (ESI) available: Experimental details, synthetic procedures, additional tables and figures. See DOI: 10.1039/c7sc01277b



**DOI:** 10.1039/c7sc01277b

**Published:** 2017-05-04

**Authors:** Jane J. Leung, Julien Warnan, Dong Heon Nam, Jenny Z. Zhang, Janina Willkomm, Erwin Reisner

**Affiliations:** a Christian Doppler Laboratory for Sustainable SynGas Chemistry , Department of Chemistry , University of Cambridge , Lensfield Road , Cambridge CB2 1EW , UK . Email: reisner@ch.cam.ac.uk ; http://www-reisner.ch.cam.ac.uk

## Abstract

The development of photoelectrodes capable of light-driven hydrogen evolution from water is an important approach for the storage of solar energy in the form of a chemical energy carrier. However, molecular catalyst-based photocathodes remain scarcely reported and typically suffer from low efficiencies and/or stabilities due to inadequate strategies for interfacing the molecular component with the light-harvesting material. In this study, we report the straightforward preparation of a p-silicon|mesoporous titania|molecular catalyst photocathode assembly that is active towards proton reduction in aqueous media with an onset potential of +0.4 V *vs.* RHE. The mesoporous TiO_2_ scaffold acts as an electron shuttle between the silicon and the catalyst, while also stabilising the silicon from passivation and enabling a high loading of molecular catalysts (>30 nmol (geometrical cm)^–2^). When a Ni bis(diphosphine)-based catalyst is anchored on the surface of the electrode, a high turnover number of ∼1 × 10^3^ was obtained from photoelectrolysis under UV-filtered simulated solar irradiation at 1 Sun after 24 h at pH 4.5. Notwithstanding its aptitude for molecular catalyst immobilisation, the p-Si|TiO_2_ photoelectrode showed great versatility towards different catalysts and pH conditions, with photoelectrocatalytic H_2_ generation also being achieved with platinum and a hydrogenase as catalyst, highlighting the flexible platform it represents for many potential reductive catalysis transformations.

## Abstract 2

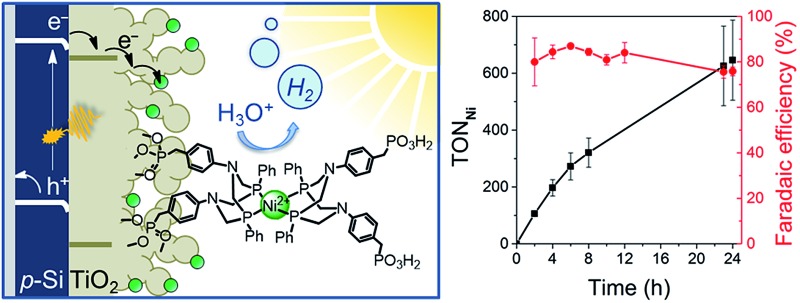
A versatile platform for the immobilisation of molecular catalysts on a readily-prepared Si photocathode with a mesoporous TiO_2_ layer is reported.

## Introduction

Inspired by natural photosynthesis, photoelectrosynthesis of solar fuels (*e.g.* H_2_ from H_2_O or carbon-based molecules from CO_2_) is of major interest in light of increasing energy demands and the depletion of non-renewable sources.^1,2^ Due to their numerous advantages – *i.e.* selective and atom-efficient catalysis, transparency, and tuneability – the use of molecular catalysts represents an interesting strategy in developing integrated systems for solar water splitting and CO_2_ reduction.^3,4^ Several approaches have been pursued to electrically wire a molecular catalyst to a light harvester, both homogeneously and heterogeneously.^5–7^ By addressing problems such as slow kinetics and the need for a high catalyst concentration, the heterogenisation of molecules has recently gained more interest, resulting in the development of molecular catalyst-based photoelectrochemical (PEC) cells.^8–19^

Despite significant progress being made in the assembly of molecular photoelectrodes, light-driven, H_2_-evolving, molecular-based photocathodes that operate in aqueous media remain scarcely reported (Table S1†).^16,20–23^ Those reported frequently suffer from low photocurrents, tedious optimisations, complex electrode architectures, modest photo-stabilities and limited versatility towards different molecular catalysts.^11,19^ In this context, the straightforward and robust combination of molecular catalysts with a light-harvesting surface remains a major challenge, due in part to the need for water-stable light harvesters and molecular catalysts. Furthermore, a functional and efficient device requires the two components to be paired in a way to allow for effective electronic communication, whilst maintaining their intrinsic physicochemical properties, and providing a high loading of the catalyst.

Silicon is the second-most earth-abundant element in the Earth's crust and its widespread utilisation in the photovoltaic industry has resulted in a substantial price drop for crystalline Si in recent years.^24^ In addition, it possesses a conduction band (CB) energy level of around –0.5 V *vs.* NHE and a band gap (*E*_g_) of 1.12 eV. This categorises Si as a potentially promising material for the assembly of a photoelectrode with significant driving force for proton reduction and the ability to harvest photons across a wide range of wavelengths, even those in the infrared.^25,26^ Impressive photocurrents for proton reduction have been previously observed when p-doped Si (p-Si) was paired with a non-molecular catalyst.^27–32^ Unfortunately, owing to the material's instability in aqueous or aerobic conditions due to the formation of an insulating silica (SiO_*x*_) layer, these currents were not always maintained. Perhaps for this reason, proton reduction by immobilised molecular catalysts on p-Si has so far only been achieved in organic solvents.^12,17^ Different protection layers have been reported to limit this instability, but often require severe precautions and expensive techniques during fabrication, such as atomic layer deposition (ALD) or vacuum-driven deposition methods.^31^

Another potential limitation to the implementation of commercial p-Si as a light-harvesting substrate in photocathodes is its inherent flatness. This is especially problematic for molecular catalysts as they typically turn over more slowly than the benchmark noble metal platinum and have a larger footprint, which requires an increased loading capacity to compensate for the reduced per effective surface area activity on the photocathode. A similar problem is addressed in dye-sensitised solar cells (DSSCs), where a high surface area architecture, commonly a mesoporous TiO_2_ layer, is employed to boost the loading capacity of molecular dyes.^33,34^ Incidentally, owing to its metal oxide nature and hydrophilicity, TiO_2_ has been extensively reported as a tolerant, functionalisable platform for the immobilisation of a wide range of chemical species.^34–36^ Due to its conduction and valence band energy levels being both lower than those of p-Si, TiO_2_ is also expected to be able to act as an electron-selective layer that shuttles electrons to a surface-immobilised catalyst. Finally, TiO_2_ has been shown to be able to protect p-Si as a flat ALD-deposited layer.^17,27^

**Fig. 1:**
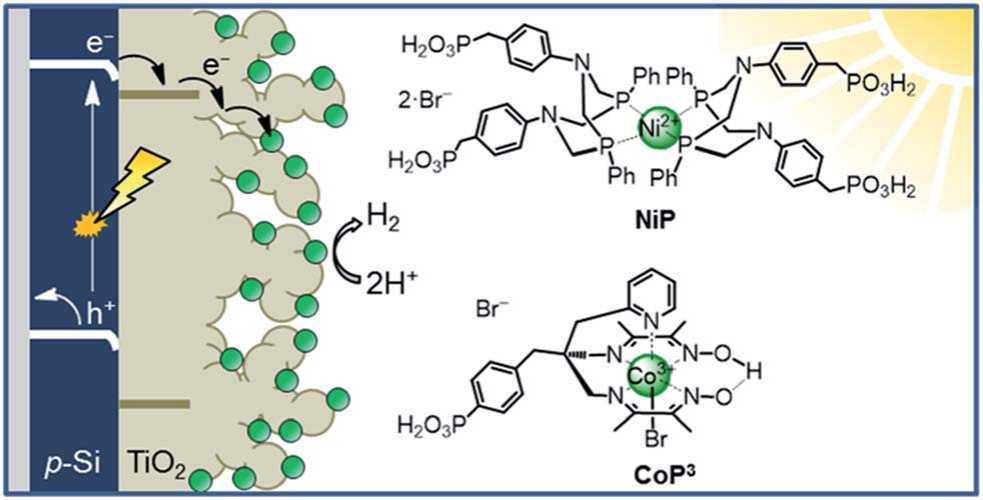
Schematic diagram of PEC H_2_ evolution with the Si|mesoTiO_2_|catalyst photocathode and chemical structures of the immobilised catalysts **NiP** and **CoP^3^**.

This study aims to demonstrate that a molecular catalyst can be efficiently and straightforwardly interfaced with p-Si to photoelectrochemically produce molecular hydrogen in aqueous conditions. Concurrently, we sought to engineer versatility in the choice of catalyst by employing a functionalisable mesoporous titanium dioxide (mesoTiO_2_) interlayer and exploiting the respectable open-circuit voltage achievable with p-Si. To this end, we anchored two phosphonated molecular proton reduction catalysts developed previously in our laboratory, DuBois-type **NiP** and cobalt diimine-dioxime **CoP^3^**, at the surface surface of a mesoporous TiO_2_ scaffold slot-coated onto p-Si (Fig. 1).^37,38^ We also used metallic platinum and a [NiFeSe]-hydrogenase (H_2_ase; Fig. S1†) as proton reduction catalysts, with the former acting as a benchmark precious metal and the latter determining the photocathode's biocompatibility. Finally, we used PEC studies to characterise the activity and stability of these electrodes, and explored the presence of long-lived charges in the CB of TiO_2_.

## Results and discussion

### Assembly of molecular photocathodes

**Fig. 2:**
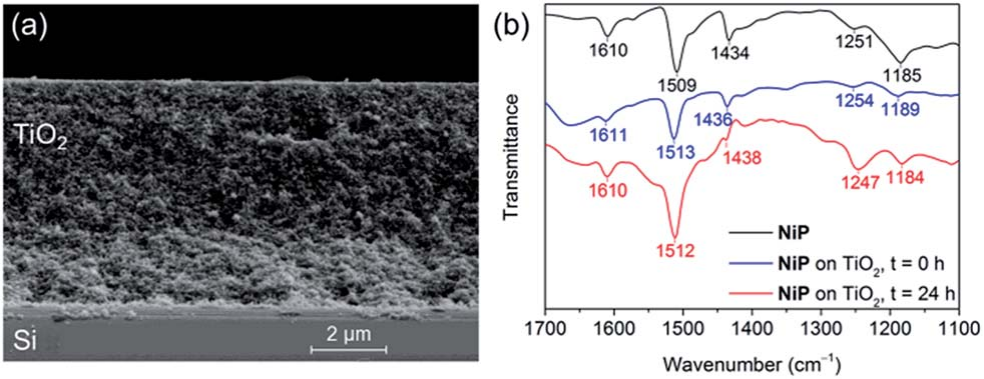
(a) SEM cross-section image of a Si|mesoTiO_2_ electrode. (b) ATR-FTIR spectra of **NiP** (black), Si|mesoTiO_2_|**NiP** before (blue) and after (red) 24 h CPP.

The preparation of a stabilising mesoporous TiO_2_ scaffold on p-Si was achieved in a relatively straightforward manner. Immediately after etching p-Si with hydrofluoric acid, a TiO_2_ paste (15–20 nm particles, 100% anatase) was slot-coated over a defined area of p-Si and the assembly annealed under atmospheric conditions following a controlled sintering procedure up to 450 °C. The approximately 6 micron-thick TiO_2_ layer of the resulting electrode (Si|mesoTiO_2_) is homogeneous and crack-free (see scanning electron microscopy, SEM, images in Fig. 2a and S2†). The uniformity of the mesoTiO_2_ film maintains semi-transparency (Fig. S3†), a light-scattering-limiting and antireflective property that allows p-Si to collect more of the incident photons during front illumination.

The phosphonated **NiP** belongs to a family of hydrogenase-inspired Ni(ii) bis(diphosphine) H_2_ evolution catalysts that display high activity and operate in both aqueous and non-aqueous conditions.^37,39,40^ The presence of phosphonic acid groups allows for robust binding to metal oxides, which makes **NiP** a promising candidate for single-site heterogeneous proton reduction on electrodes. In addition, the Co diimine–dioxime catalyst **CoP^3^** was used as a proton reduction catalyst. **CoP^3^** bears a phosphonic acid anchoring group covalently bonded to the equatorial ligand of the catalyst core for robust attachment on metal oxides, and a pendant axial pyridine ligand to improve the performance for H_2_ catalysis.^38^ Despite the high loading of these molecules on metal oxides having been previously demonstrated, their successful incorporation as functional catalysts in a photocathodic device remains elusive.^18,38^

Immobilisation of the molecular catalysts **NiP** or **CoP^3^** was accomplished *via* overnight immersion of Si|mesoTiO_2_ electrodes in a methanol (MeOH) solution of the catalyst (0.25 mM) to yield the final Si|mesoTiO_2_|**NiP** and Si|mesoTiO_2_|**CoP^3^** photocathodes, respectively. At this point, clear colour changes of the mesoTiO_2_ scaffold that correspond to the original colours of the molecular catalysts (yellow in the case of Si|mesoTiO_2_|**CoP^3^** and purple for Si|mesoTiO_2_|**NiP**|; Fig. S3†) testify to their successful immobilisation. Pt was thermodeposited on Si|mesoTiO_2_ from a solution of hexachloroplatinic acid, resulting in a transparent Si|mesoTiO_2_|Pt electrode. Full experimental details are given in the ESI.†

Attenuated total reflectance Fourier-transform infrared (ATR-FTIR) spectroscopy experiments confirmed the catalysts' successful attachment to TiO_2_ through the comparison of the spectra of the unbound catalysts to that of their corresponding catalyst-loaded electrodes. Consistent with the ATR-FTIR spectrum obtained of the **NiP** powder, vibration bands at 1610, 1509 and 1434 cm^–1^ were also observed in the spectrum of Si|mesoTiO_2_|**NiP** and were attributed to the aromatic rings' *ν*(C=C) and the *δ*(C–H) of the methylene bridges (Fig. 2b, blue trace). In the case of **CoP^3^**-functionalised Si|mesoTiO_2_ electrodes, the aromatic rings' *ν*(C=C) and *ν*(C=N) were recorded at 1617 and 1538 cm^–1^ (Fig. S4†).

X-ray photoelectron spectroscopy (XPS) spectra show characteristic binding energy peaks in the Co_2p_ or Ni_2p_, N_1s_ and P_2p_ regions for fresh Si|mesoTiO_2_|**CoP^3^** and Si|mesoTiO_2_|**NiP** electrodes, respectively, at energies close to those previously reported for similar catalysts (Fig. S5–S6†).^18,41,42^ In the Co_2p_ region of the former, two broad signals corresponding to 2p_1/2_ and 2p_3/2_ core levels were observed at 795.4 and 780.4 eV respectively, whereas the Ni_2p_ region of the latter shows the same respective core levels at 872.0 and 854.8 eV. Peaks in the N_1s_ and P_2p_ core level regions of both photocathodes arise from their ligands and anchoring groups.

The amount of **CoP^3^** and **NiP** loaded onto Si|mesoTiO_2_ electrodes was quantified by spectrophotometry following desorption of the catalyst from the corresponding electrode with tetrabutylammonium hydroxide in MeOH (0.1 M; Fig. S7†). The loading of **CoP^3^** and **NiP** on Si|mesoTiO_2_ was determined as 93.9 ± 8.9 nmol cm^–2^ and 38.3 ± 4.2 nmol cm^–2^ (geometric surface area), respectively (Table S2†). The higher loading of **CoP^3^** is in line with its smaller steric footprint as compared to the **NiP** molecule. These numbers are consistent with previously reported loadings onto mesoporous metal oxide-based electrodes for phosphonic acid-bearing catalysts.^15,18,38^

### Photoelectrocatalytic H_2_ generation

**Fig. 3:**
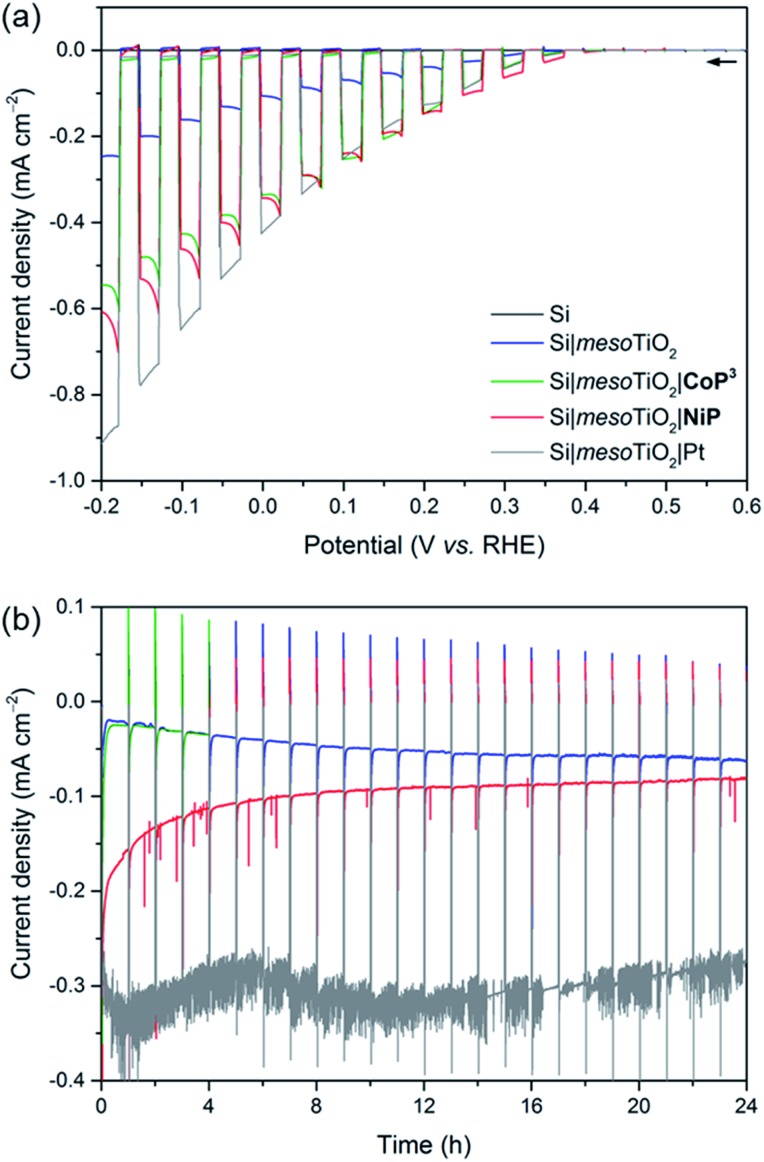
(a) LSVs of Si (black), Si|mesoTiO_2_ (blue), Si|mesoTiO_2_|**CoP^3^** (green), Si|mesoTiO_2_|**NiP** (red) and Si|mesoTiO_2_|Pt (grey) electrodes under chopped illumination (*υ* = 5 mV s^–1^). (b) CPP traces for Si|mesoTiO_2_, Si|mesoTiO_2_|**NiP**, Si|mesoTiO_2_|**CoP^3^** and Si|mesoTiO_2_|Pt electrodes (see (a) for colour-coding) at *E*_app_ = 0.0 V *vs.* RHE under continuous illumination with an hourly dark chop lasting for two min each. Conditions: aqueous acetic acid solution (0.1 M, pH 4.5), UV-filtered simulated solar light irradiation (AM1.5G, 100 mW cm^–2^, *λ* > 400 nm), N_2_ atmosphere, room temperature.

Linear sweep voltammograms (LSVs) under chopped UV-filtered AM1.5G illumination were recorded of the Si|mesoTiO_2_|**NiP** and Si|mesoTiO_2_|**CoP^3^** photocathodes and compared to those of Si|mesoTiO_2_|Pt, Si|mesoTiO_2_ and bare Si (submitted to the same sintering steps as all other electrodes; Fig. 3a). A photocurrent onset potential (*E*_onset_) of approximately 0.4 V *vs.* RHE is observed for all Si|mesoTiO_2_ electrodes (with and without catalysts), and the TiO_2_-free (bare) p-Si counterpart remains inactive even at more cathodic potentials. The passivity of the bare p-Si electrode is attributed to the fast, heat-accelerated formation of an insulating layer of silica *via* a thermal oxidation reaction, which is consistent with previous reports.^43^ A large proportion of the photocurrent (*J*) observed from the catalyst-free Si|mesoTiO_2_ electrode originates from a “charging” current (filling of the metal oxide's CB) and this mechanism is discussed in more detail below. It is noteworthy that simple slot-coating with a porous TiO_2_ scaffold limits complete Si insulation and enables a productive electron transfer pathway from photoexcited p-Si to the CB of TiO_2_, despite the high temperature annealing process under atmospheric conditions.

The photocurrent is enhanced upon loading of Si|mesoTiO_2_ with proton reduction catalysts: at 0.0 V *vs.* RHE, a photocurrent of –430 μA cm^–2^ is obtained with Si|mesoTiO_2_|Pt, whereas Si|mesoTiO_2_|**NiP** and Si|mesoTiO_2_|**CoP^3^** achieve approximately –340 μA cm^–2^. The broad cathodic recombination peaks observed in the LSVs of the molecular catalyst-loaded electrodes might originate from the slower kinetic rate of **NiP** and **CoP^3^** to photo-generate H_2_, as compared to Pt. All catalyst-modified electrodes exhibit slightly earlier onset potentials than the unmodified Si|mesoTiO_2_ electrode (Fig. S8†). Nevertheless, the proximity of these values across all electrodes suggests that *E*_onset_ is predominantly controlled by the p–n Si–mesoTiO_2_ interface, irrespective of modifications at the TiO_2_–electrolyte interface.^17^

Although *E*_onset_ compares well with previously reported crystalline p-Si-based photocathodes,^28,44^ we observed a relatively small photocurrent with Si|mesoTiO_2_|Pt,^31^ which may be attributed to the formation of some insulating SiO_*x*_ layer during the aerobic sintering process. Nonetheless, our results with Si|mesoTiO_2_|Pt confirm that electron transfer from p-Si to a TiO_2_-bound proton reduction catalyst is possible, and simultaneously allow us to elucidate the maximum photocurrent that is likely to be obtainable with our photocathode preparation if kinetic barriers did not exist at the catalyst–electrolyte interface. In fact, the photocurrent densities obtained compare well with those of the molecular catalyst-based electrodes, indicating that the molecular-based electrodes perform at maximum performance that can be expected under these conditions. The effects of different mesoTiO_2_ thicknesses and pH conditions were also studied for the Si|mesoTiO_2_|**NiP** photocathodes (Fig. S9†). Reducing the thickness of the mesoporous scaffold resulted in a proportionately lower loading of **NiP** (5.6 ± 1.4 nmol cm^–2^ on mesoTiO_2_ with a thickness of 1.1 μm, Table S2†) and consequently gave rise to a lower performing photocathode (Fig. S9a†). A pH optimum was observed at 4.5, which agrees with previous catalytic studies with **NiP** (Fig. S9b†).^37^

**Fig. 4:**
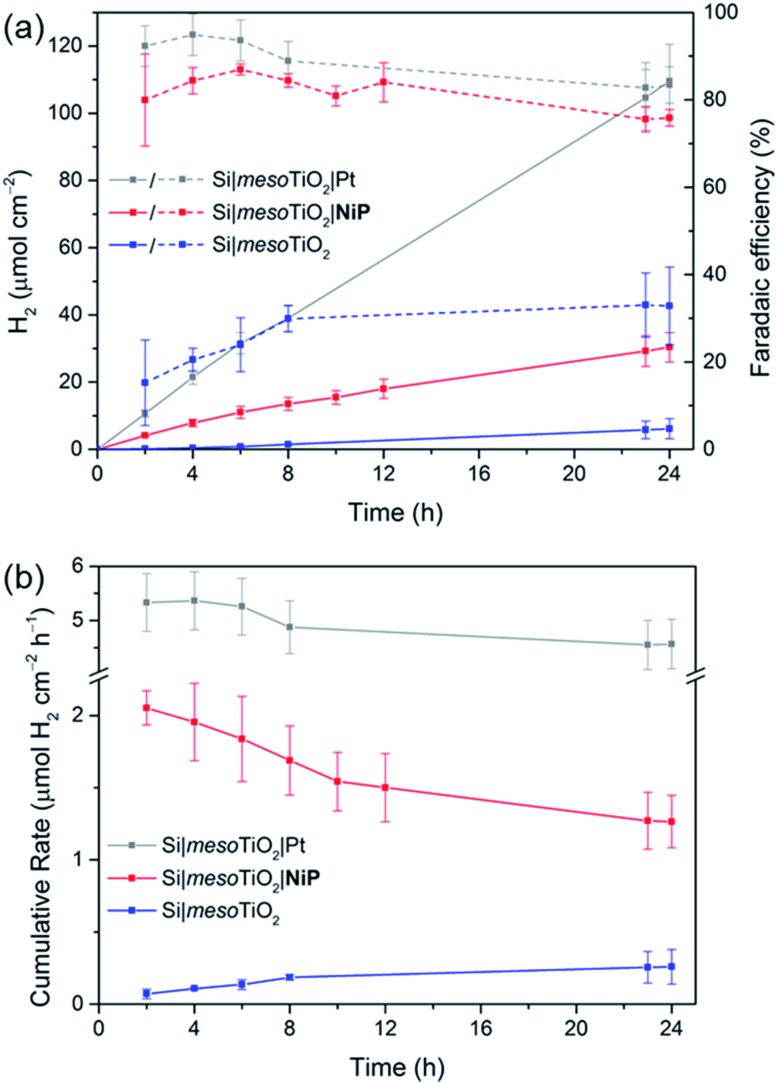
CPP data for Si|mesoTiO_2_ (blue trace), Si|mesoTiO_2_|**NiP** (red trace) and Si|mesoTiO_2_|Pt (grey trace) electrodes at 0.0 V *vs.* RHE during continuous UV-filtered simulated solar light irradiation (AM1.5G, 100 mW cm^–2^, *λ* > 400 nm). (a) H_2_ evolution (solid lines) and cumulative faradaic efficiency (dashed lines); (b) cumulative rate of H_2_ production per geometrical surface area of the electrodes. Conditions: aqueous acetic acid solution (0.1 M, pH 4.5), inert atmosphere, room temperature.

Having established the photocathode architecture as a viable platform on which to interface molecular catalysts, their prolonged H_2_ evolution performance and stability were studied. Controlled potential photoelectrolysis (CPP) under UV-filtered simulated solar light illumination at an applied potential (*E*_app_) of 0.0 V *vs.* RHE (Fig. 3b) was employed for 24 h and the headspace H_2_ was analysed with gas chromatography at regular intervals (Fig. 4a). The bare p-Si electrode produced only a miniscule photocurrent density and detectable amounts of H_2_ were not observed (Fig. S10†). Although the Si|mesoTiO_2_ control electrode is not innocent in proton reduction and produces a small amount of H_2_, its low faradaic efficiency (FE) of 28–30% shows that the majority of the photocurrent is generated from other processes, such as charging the CB of TiO_2_ (see discussion below). Si|mesoTiO_2_|**NiP** displays a FE up to 87% in the early stages of CPP and remains at 76 ± 2% after 24 h. An increasing FE was observed during the first few hours, which is likely due to initial filling of trap states in TiO_2_ and the reduction of residual O_2_ in the pores of the mesoporous scaffold.^35^ Generally, the slightly lower FE compared to the Pt-based electrode could be attributed to the progressive degradation/desorption of some reduced **NiP**, leading to unproductive electron transfer pathways. Nevertheless, these values are consistent with the FE reported previously for dark electrolysis of **NiP** on a TiO_2_ electrode.^18^ Tracking the cumulative rate of H_2_ production per surface area also shows that the **NiP**-modified photocathode continues to exhibit greater H_2_ evolution activity compared to an unmodified Si|mesoTiO_2_ electrode even after a day (Fig. 4b).

A **NiP**-based turnover number (TON**_NiP_**) of 646 ± 141 was obtained after 24 h CPP with the Si|mesoTiO_2_|**NiP** electrodes (note that this value was corrected for H_2_ evolution from Si|mesoTiO_2_ and is therefore a lower estimate of the true activity; see Fig. S11†). This catalytic performance is in agreement with previously reported TON**_NiP_**, where **NiP** was used in electrocatalytic and photocatalytic H_2_ generation with sacrificial electron donors.^18,37,45^ This observation therefore demonstrates that the intrinsic activity of **NiP** can be exploited upon immobilisation onto photoelectrodes and supports that the catalyst's molecular integrity is maintained throughout CPP, as opposed to the previously reported transformation of some immobilised molecular catalysts under catalysis conditions.^46–49^

Si|mesoTiO_2_|**NiP** achieved an initial photocurrent of ∼–210 μA cm^–2^, which drops to approximately half after 8 hours. The photocurrent loss is likely due to slow desorption of the catalyst from the mesoTiO_2_ scaffold and/or its progressive degradation.^45,50^ While the slow deactivation of Si|mesoTiO_2_|**NiP** is apparent through both its gradually decreasing FE and rate of H_2_ production, Si|mesoTiO_2_|Pt, on the other hand, continues to evolve H_2_ at near unity FE even after 24 h of CPP, maintaining a steady photocurrent density throughout (Fig. 4). The precious metal catalyst thereby demonstrates the enduring stability of the Si|mesoTiO_2_ architecture in aqueous conditions, and highlights its appeal as a scaffold for different proton reduction catalysts across a range of stabilities.

CPP of Si|mesoTiO_2_|**CoP^3^** reveals a photocathode that also possesses proton reduction capabilities superior to that of the unmodified Si|mesoTiO_2_ electrode for up to 1 h (Fig. 3b and S12†). A progressive decline in the photocathode's performance is, however, apparent in both its slowing H_2_ production rate and decreasing photocurrent density. This trend is in agreement with previous reports of the limited stability of **CoP^3^**, where degradation may be attributed to ligand hydrogenation and/or the formation of a ligand radical species.^38,51–53^ A final TON**_CoP3_** of 10.5 ± 0.5 (background H_2_ evolution from Si|mesoTiO_2_ subtracted) was achieved after 4 h.

The incident photon-to-current efficiency (IPCE) spectrum of Si|mesoTiO_2_|**NiP** at *E*_app_ = 0.0 V *vs.* RHE showed approximately 6% across all measured wavelengths (450–850 nm) and an approximately two-fold enhancement compared to that of Si|mesoTiO_2_ (Fig. S13†). The photoresponse covers the visible and extends into the IR region, maintaining relative homogeneity across the wavelengths, and highlights the clear advantage brought about by p-Si compared to other light absorbers as it allows the conversion of low-energy photons into free charge carriers for the reduction of protons to H_2_.

### Post-catalysis characterisation and molecular integrity

Post-catalysis characterisation of molecular catalyst-based photocathodes is important to assess the molecular integrity in a field where the transformation of molecular catalysts into non-molecular active species is a constant possibility.^46,48,49,54^ The gradual decrease in catalytic activity over time as well as features in ATR-FTIR and XPS spectra support the lasting integrity of the molecular **NiP** catalyst on Si|mesoTiO_2_|**NiP**, greatly limiting the possibility that the enduring activity from this photocathode arises from the formation of some other non-molecular Ni-based catalytic species. All characteristic bands across the ATR-FTIR spectrum of the photocathode prior to electrolysis reappear in the spectrum of the photocathode that has undergone a full day of CPP (Fig. 2b). In addition, all binding energy peaks arising from core levels in the Ni_2p_, N_1s_ and P_2p_ regions are present in the spectra of both fresh and electrolysed photocathodes, and metallic Ni (852.6 eV) that might have been suspected to contribute to catalysis was not detected (Fig. S6†). The reduced intensity of these XPS peaks after electrolysis is likely attributable to the initial rapid loss of catalyst molecules chemisorbed on the top of the TiO_2_, whereas molecules trapped deeper within the porous matrix give rise to catalytic activity over a longer period of time but are not readily accessible by XPS. Finally, comparing LSVs of the Si|mesoTiO_2_|**NiP** photocathode both before and after 24 h of photoelectrolysis shows the retention of broad cathodic recombination peaks characteristic of molecular catalysts' slow kinetics, along with a decrease in photocurrent intensity by roughly half (Fig. S14†). This is in agreement with some loss/degradation of the molecular catalyst from the photocathode, as previously deduced from the chronoamperogram.

In contrast, both ATR-FTIR and XPS analysis confirm the instability of **CoP^3^** on the Si|mesoTiO_2_|**CoP^3^** photocathode (Fig. S4 and S5†). Characteristic IR bands and Co_2p_ and P_2p_ signals in the XPS spectra have mostly disappeared or are altered after CPP, leaving behind only a weak N_1s_ XPS signal, probably as a result of small traces of ligand species still attached to the surface. These results further highlight the impressive durability of **NiP** in a field where stable, highly active immobilised molecular catalysts for the hydrogen evolution reaction remain hard to identify, especially on photocathodes.

### Biocompatibility of Si|mesoTiO_2_ photocathode

The versatility of our Si|mesoTiO_2_ cathodes was further demonstrated by interfacing them with a [NiFeSe]-H_2_ase from *Desulfomicrobium baculatum* as the proton reduction catalyst.^55^ H_2_ases display exceptionally high catalytic rates for H_2_ production at marginal overpotentials that are currently only matched by platinum. [NiFeSe]-H_2_ases are particularly suitable catalysts for applications in water splitting as they display a bias towards H_2_ evolution in the presence of O_2_, with marginal inhibition by H_2_.^56^ The immobilisation of the H_2_ase onto our electrodes was achieved by drop-coating a solution of 8 pmol of the enzyme on Si|mesoTiO_2_ under an inert atmosphere, affording the Si|mesoTiO_2_|H_2_ase electrode. The interaction between TiO_2_ and H_2_ase is thought to occur at the surface-exposed glutamate and aspartate residues in close proximity to the distal Fe–S cluster relay, the latter of which is expected to deliver photo-excited electrons to the embedded active site for H_2_ generation.^57,58^ LSVs revealed a clear enhancement of the unmodified Si|mesoTiO_2_'s photocurrent upon introduction of the enzyme, with *J* = –195 μA cm^–2^ achieved at 0.0 V *vs.* RHE for Si|mesoTiO_2_|H_2_ase (Fig. S15†).

**Fig. 5:**
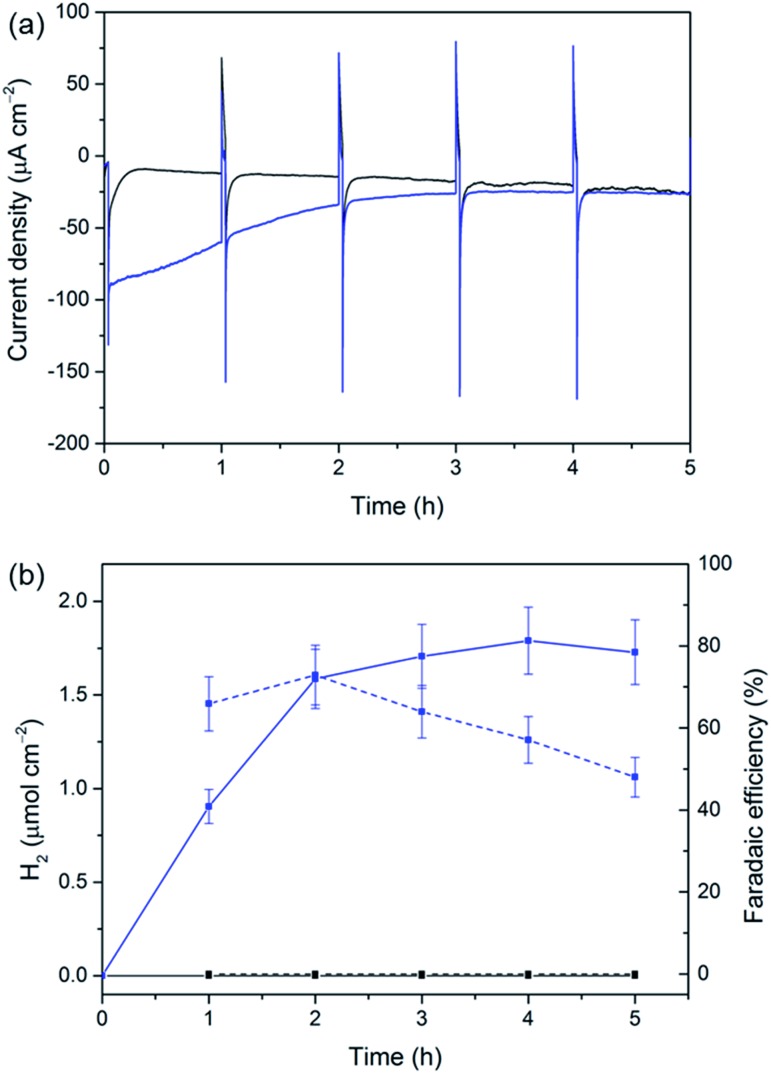
CPP traces for Si|mesoTiO_2_ (black) and Si|mesoTiO_2_|H_2_ase (blue) at 0.0 V *vs.* RHE during continuous illumination with UV-filtered simulated solar light (AM1.5G, 100 mW cm^–2^, *λ* > 400 nm). (a) Chronoamperograms with an hourly dark chop lasting for two min each and (b) H_2_ evolution (solid traces) and cumulative faradaic efficiency (dashed traces). Conditions: aqueous 2-(*N*-morpholino)ethanesulphonic acid solution (50 mM, pH 6), inert atmosphere, room temperature.

Si|mesoTiO_2_|H_2_ase displays a significantly higher initial photocurrent density (–89.7 μA cm^–2^, Fig. 5a) and rate of H_2_ production (Fig. 5b) during sustained CPP at 0.0 V *vs.* RHE than the catalyst-free Si|mesoTiO_2_ electrode under identical conditions. These observed photocurrents exceed those previously reported for a Si|flat–TiO_2_|H_2_ase by a factor of 30,^59^ highlighting the advantages of a homogeneous and well-connected mesoporous-structured TiO_2_ scaffold.

We note that the large footprint of the H_2_ase (8–10 nm)^60^ makes full penetration of the enzyme throughout the mesoporous TiO_2_ film (pore size ≈ 15–20 nm) difficult and it is likely that the enzyme has adsorbed mostly on top of the mesoporous scaffold, leaving a significant proportion of TiO_2_ beneath unmodified. Un-optimal coverage due to an inefficient penetration depth of the enzyme is therefore a likely explanation for the less-than-unity FE and limited lifetime of ∼4 h for Si|mesoTiO_2_|H_2_ase during CPP, after which ‘film loss’ (degradation, re-orientation or desorption) has removed the electroactive enzyme film. Nevertheless, the biocompatibility of TiO_2_ withholds this metal oxide as an attractive interfacing material on which to achieve effective adsorption of enzymes. Work is currently underway to rationally design a photocathode that maintains the merits of the Si|mesoTiO_2_ interface whilst optimising the scaffold's dimensions to better accommodate large biomolecules like hydrogenase.

### Comparison with the state-of-the-art

A challenge in the preparation of molecular catalyst-based photocathodes is the transferral of the catalyst's solution performance onto a robust photoelectrode. This difficulty is illustrated by the low turnover numbers (<3) in recent attempts to integrate **NiP** onto photocathodic architectures.^13,14^ With a TON**_NiP_** value >600, our system achieves catalytic cycles that are among the highest reported for a molecular catalyst on a photocathode and in the same order of magnitude as previously-reported solution systems.^37,45,61^ In addition, we achieved a TON**_CoP3_** of >10 with **CoP^3^**, which is also consistent with the previously reported performance of this catalyst during solar H_2_ production on dye-sensitised TiO_2_ nanoparticles in the presence of a sacrificial hole scavenger.^38^ These results, together with the demonstrated biocompatibility of the Si|mesoTiO_2_ electrode, show a chemical benignancy of the photocathode assembly towards a variety of catalysts and thus highlight the potential adaptability. In addition, the assessed stability and molecular nature of the Si|mesoTiO_2_|**NiP** electrode and **NiP** catalyst, respectively, reflect the congruity of the system in a field where TONs are scarcely reported and sometimes remain subject to fundamental questions regarding molecular integrity.^48,62^

Another peculiar problem lies in designing an efficient and simple interface to integrate the molecular catalyst with the light-harvesting material. Unlike previously reported systems where the catalyst has been immobilised alongside a dye on a porous metal oxide (*e.g.* NiO),^13,14,22,23,63–66^ or deposited at the surface of a flat photoactive material,^16,20,21,67–71^ our system separates the light harvester from the catalyst *via* a mesoporous n-type semiconductor layer, which presents several benefits. As demonstrated by major advances realised in its preparation over the past years, such a mesoporous TiO_2_-based interlayer could deliver a straightforward, generalisable and high surface area catalyst immobilisation platform *via* popular anchoring groups.^15,33^ Concomitantly, it affords a direct, fast electron transfer to the bound catalyst as a result of the existence of a chemical bond, as well as low probabilities of charge recombination between the catalyst and the light harvester by acting as a hole-blocking layer. Despite all of its above advantages, a mesoporous TiO_2_ scaffold had not yet been employed in a molecular photocathode for PEC H_2_ evolution; under aqueous conditions; nor without the need for an additional ALD-deposited interlayer. The presence of a mesoTiO_2_ interlayer in our system does not strongly affect the potential photovoltage of the silicon electrode, as the measured underpotential for proton reduction (≈0.4 V *vs.* RHE) compares well with those of previously reported p-Si-based photocathodes.^27,30^ Finally, in contrast to NiO-based photocathodes, the system does not require any other co-immobilised molecules due to its light harvester|mesoTiO_2_|catalyst architecture, thereby avoiding time-consuming ratio optimisations, kinetic and stability limitations resulting from using an added dye. Thus, the photocathode displayed a photocurrent of –340 μA cm^–2^ at 0.0 V *vs.* RHE when loaded with **NiP**, representing a 3- to 150-fold improvement as compared to the results reported with dye-sensitised NiO-based architectures (Table S1†).

Although the photocurrents achieved with our molecular-based photocathodes are relatively modest compared to the best state-of-the-art photocathodes (Table S1†), similar values are attained with the platinised equivalent. This shows that our system's bottleneck probably originates from the limited number of available charge carriers and that it could therefore be improved by optimising the electrode preparation procedure. On the other hand, the modest photovoltage displayed by the Si|mesoTiO_2_|catalyst architecture has its main origin in p-Si's small band gap and charge recombination.

Nevertheless, as confirmed by the IPCE measurements, p-Si allows for the broadest conversion of wavelengths, including low-energy IR photons (EQE = 7% at 850 nm), among molecular catalyst-based electrodes. Consequently, such an architecture would benefit the preparation of a molecular-based tandem PEC device towards full water splitting when utilised as the proton-reducing electrode.

### Analysis of TiO_2_ charging currents

**Fig. 6:**
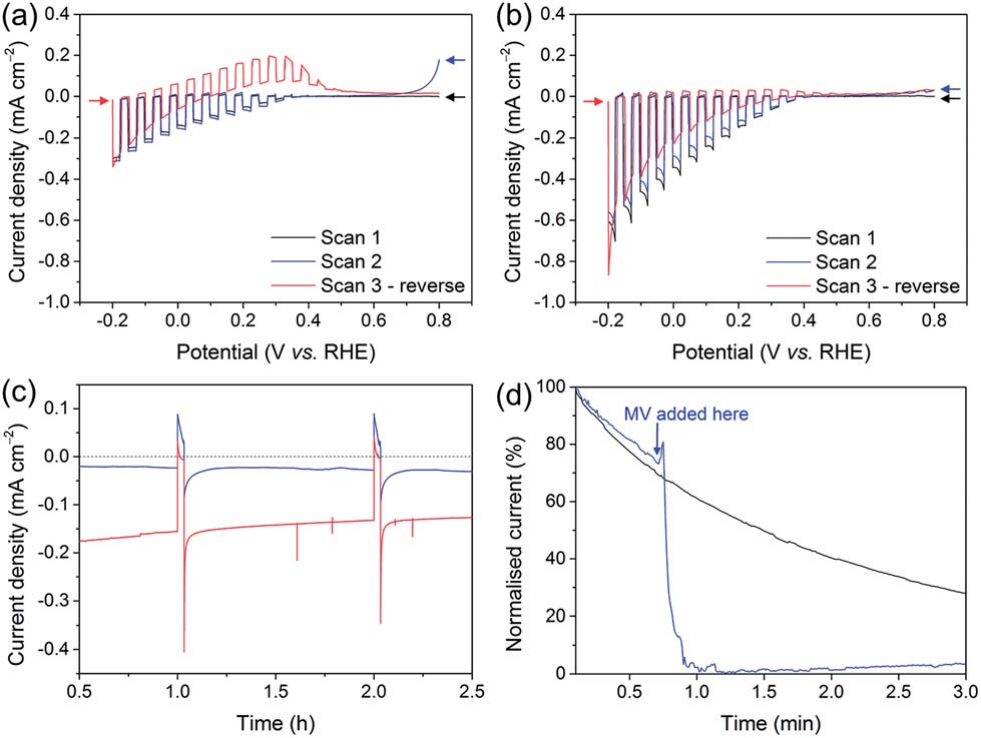
LSVs under chopped illumination of (a) Si|mesoTiO_2_ and (b) Si|mesoTiO_2_|**NiP** with the first two scans in cathodic direction and the third scan in anodic direction following scan reversal. Arrows indicate scanning direction for all LSVs (*υ* = 5 mV s^–1^). (c) Enlarged view of the first two dark chops during CPP of Si|mesoTiO_2_ (blue) and Si|mesoTiO_2_|**NiP** (red). (d) Chronoamperogram of Si|mesoTiO_2_ in the dark (normalised and given as a percentage) after being exposed to light (to charge the CB), without MV (black trace) and with MV added partway (blue trace); *E*_app_ = 0.0 V *vs.* RHE. Conditions: aqueous acetic acid solution (0.1 M, pH 4.5), UV-filtered simulated solar light irradiation (AM1.5G, 100 mW cm^–2^, *λ* > 400 nm), inert atmosphere, room temperature.

The development of the Si–mesoTiO_2_ interface led us to study the charging and discharging of the CB of TiO_2_. A charging behaviour was first evidenced when performing consecutive LSVs in both reducing and oxidising scanning directions on the photocathodes (Fig. 6a). In the case of LSVs from anodic to cathodic potentials for the catalyst-free Si|mesoTiO_2_ electrode, the second scan displays an anodic dark current at the scan start (0.8 V *vs.* RHE, blue trace). Scanning in the anodic direction upon scan reversal from the cathodic scan shows an anodic dark current from approximately 0.0 to 0.4 V *vs.* RHE (red trace). This can be attributed to the CB being subsequently “discharged” at more positive applied potentials, giving rise to an anodic dark current.

When either **NiP**, Pt or H_2_ase is surface-bound on TiO_2_, no discharging features were observed (Fig. 6b, S16a and b†). This observation signifies efficient charge transfer from the CB of TiO_2_ to the proton reduction catalyst as a mechanism of utilising CB electrons. In other words, these catalysts, even molecular **NiP**, are effective at lowering the kinetic barrier and therefore providing high activity for proton reduction. Even after 24 h of CPP, no evidence of substantial charge accumulation from charging is observed in the case of Si|mesoTiO_2_|**NiP**, which is made apparent by the lack of an anodic dark current in the reverse LSV scan (Fig. S16c,† red trace). This indicates that the amount of molecular catalyst remaining on the cathode is sufficient to ensure efficient extraction of charges from the CB of TiO_2_, and hence provides a good FE. Taking this into account and considering the clear kinetic advantage of **NiP** compared to catalyst-free TiO_2_, all electrons reaching the solution *via* the catalyst and not directly from TiO_2_ would give an upper estimate of TON**_NiP_** (24 h) = 1082 ± 244 (without background subtraction).^72^

In contrast, fresh **CoP^3^**-loaded electrodes already feature electrochemical discharging of the CB in the second cathodic LSV with a significant drop in cathodic photocurrent intensity (Fig. S16d†). In addition, the subsequent reverse scan also displays anodic dark currents close to those recorded in the case of the bare Si|mesoTiO_2_ electrode. Both observations confirm the previously reported instability of this catalyst and/or inefficient charge extraction from the CB of TiO_2_.^38^

Anodic dark currents observed when dark chopping during CPP of Si|mesoTiO_2_ confirm the temporary storage of electrons in the CB of TiO_2_ and their subsequent discharging in the dark (Fig. 6c). On the other hand, the anodic dark current is almost absent in the case of Si|mesoTiO_2_|**NiP** as the catalyst efficiently collects electrons from the TiO_2_ CB.

We monitored the CB discharging by recording the slow decay of the anodic current during a dark chronoamperometry after having previously charged the Si|mesoTiO_2_ electrode under light (Fig. 6d). The observed slow decay (∼15 min) of the anodic current indicates that our system allows for the existence of long-lived electrons trapped in TiO_2_. Electron trapping in TiO_2_ has been extensively studied and is believed to be localised in the TiO_2_ lattice as Ti^3+^ sites, but the storage of such trapped states lasting for timescales beyond microseconds has been scarcely reported.^73,74^ In contrast, the current decays almost instantly to the baseline with the introduction of an electron acceptor (*i.e.* methyl viologen dichloride, MV; *E*_(MV/MV^–^)_ ≈ –0.45 *vs.* NHE) to the electrolyte solution, concurrent with the appearance of a blue colour from the reduced methyl viologen radical at the surface of the electrode. Both observations indicate the reduction of MV species by electrons located in the TiO_2_ CB. Performing another discharge-monitoring experiment in the presence of an anchored catalyst such as **NiP** (and with no MV) results in the absence of any anodic dark current during dark chronoamperometry following CB charging by light, as the electrons are efficiently transferred to the catalyst (Fig. S17†).

We thereby demonstrate that the well-known ability of TiO_2_ to trap electrons can be exploited in our Si|mesoTiO_2_ architecture. This represents the first application of this phenomenon *via* the fabrication of a device capable of storing visible light-generated electrons on an electrode. The realisation of these long-lived electrons following photoexcitation of the Si|mesoTiO_2_ electrode enables temporal decoupling between the photo-production of the electric charge and its utilisation in the form of electricity or chemical synthesis, and is therefore an attractive means of short (solar-charged capacitors)- and long (solar fuels)-term storage of solar energy.^75–78^

## Conclusions

We have demonstrated the solar-driven reduction of aqueous protons to hydrogen by molecular catalysts immobilised on light-harvesting p-Si. This challenging task was achieved by the straightforward deposition of an interfacing mesoporous TiO_2_ scaffold, which not only affords a high loading of molecular catalyst, but also represents a low-cost method of integrating a catalyst with p-Si. The Si–mesoTiO_2_ interface consistently offers an underpotential of 400 mV for the H_2_ evolution reaction and, in the absence of a catalyst, represents an architecture that allows for the storage of solar energy in the form of trapped electrons lasting for minutes. The charging of the CB of TiO_2_ allows us to envision the use of this electrode as a short- and long-term electron-storing, light-rechargeable device.

The molecular catalyst-modified Si|mesoTiO_2_|**NiP** reached the highest photocurrent and TON known for a DuBois-type molecular catalyst on a photocathode in aqueous media, and continues to evolve hydrogen at high faradaic efficiencies even after 24 h of operation under UV-filtered simulated solar light illumination, highlighting the stability of both the Si–mesoTiO_2_ assembly and the molecular catalyst itself. IPCE studies showed the photoelectrode's ability to utilise low-energy photons and, therefore, its suitability for coupling with large band gap-based photoanodes in a potential tandem PEC device. Finally, other catalysts used in this work (metallic Pt, molecular cobaloxime **CoP^3^** and hydrogenase) are testament to the versatility offered by our approach and the opportunities it presents for evaluating a wide array of existing and future catalysts immobilised on a stable photocathode towards solar-driven hydrogen evolution and other redox transformations.
