# Supplementary material for: Photoelectrocatalytic H2 evolution in water with molecular catalysts immobilised on p-Si via a stabilising mesoporous TiO2 interlayer
Source: Chem Sci. 2017 May 4;8(7):5172–80. doi: 10.1039/c7sc01277b (PMC5618793; doi:10.1039/c7sc01277b)
Supplement: Supplementary file 1 [file SC-008-C7SC01277B-s001.pdf]

Electronic Supplementary Material (ESI) for Chemical Science.
This journal is © The Royal Society of Chemistry 2017

Electronic Supplementary Information

Photoelectrocatalytic H_2 Evolution in Water with Molecular Catalysts Immobilised on *p*-Si *via* a Stabilising Mesoporous TiO_2 Interlayer

Jane J. Leung[‡], Julien Warnan[‡], Dong Heon Nam, Jenny Z. Zhang, Janina Willkomm, Erwin Reisner*

Christian Doppler Laboratory for Sustainable SynGas Chemistry, Department of Chemistry University of Cambridge, Lensfield Road, Cambridge CB2 1EW, United-Kingdom

E-mail: reisner@ch.cam.ac.uk

[‡]These authors contributed equally.

Contents

Experimental Section Page S2
Supporting Tables and References Page S5
Supporting Figures Page S8

Experimental Section

Si and Si|*meso*TiO_2 Electrode Fabrication

Commercial boron-doped Si wafers (1.2 cm × 2.5 cm, University wafer) were sequentially cleaned with acetone, isopropanol, ethanol, and piranha solutions for 10 min each. The bare *p*-Si electrodes were obtained after removing the native oxide layers from the surface by immersing the electrodes in hydrofluoric acid (65 %, Merck Millipore) for 1 min and then rinsing with Milli-Q® water. The mesoporous TiO_2 scaffold was deposited on *p*-Si by slot-coating commercial Ti-Nanoxide pastes (15-20 nm particles, 100 % anatase, Solaronix) over a defined area (0.7 cm × 0.7 cm). The electrodes were then sintered in a Carbolite furnace, under atmospheric conditions using the following heating ramp. Immediately after slot-coating, the electrodes were transferred into a furnace pre-heated at 135 °C. The temperature was increased to 325 °C and held at this temperature for 5 min, after which the temperature was further augmented to 375 °C and maintained for 5 min at this temperature. The temperature was finally allowed to reach 450 °C. The electrodes were removed from the furnace after a slow cooling period. The thickness of the resulting TiO_2 layers was measured by scanning electron microscopy. Layers of 6 µm or 1.1 µm were obtained when using T/SP or T600 TiO_2 pastes, respectively.

Catalyst Immobilisation and Back Contact Assembly on Si|*meso*TiO_2

Immobilisation of the molecular catalysts on Si|*meso*TiO_2 electrodes to yield Si|*meso*TiO_2|**NiP** and Si|*meso*TiO_2|**CoP^3** was carried out by soaking the electrodes in a 0.25 mM solution of the corresponding catalyst in distilled MeOH for 16 h, after which they were rinsed with MeOH. **NiP** and **CoP^3** were both synthesised according to previously reported procedures.[1,2] Platinisation of Si|*meso*TiO_2 to yield Si|*meso*TiO_2|Pt electrodes was conducted by brushing a solution of hexachloroplatinic acid hydrate in isopropanol (2 mg ml^{-1}) across the top of the electrode. This operation was repeated three times, allowing the solvent to dry between each deposition. The cells were then transferred into a furnace at 350 °C for 20 min, followed by slow cooling to room temperature. Immobilisation of H_2ase on Si|*meso*TiO_2 electrodes was carried out by first diluting a stock solution of H_2ase with 20 mM Tris/HCl buffer (pH 7) in an anaerobic glovebox to give 8 µM H_2ase aliquots (stored at −30 °C), which were thawed immediately before use.

The [NiFeSe]-hydrogenase used in this study was isolated from *Desulfomicrobium baculatum*, and was provided by Dr Juan C. Fontecilla-Camps and Dr Christine Cavazza (Institut de Biologie Structurale, Grenoble, France); it was purified using a previously published method[3] and the stock solution has a specific activity of 2115 µmol H_2 min^{-1} mg^{-1}. Prior to enzyme immobilisation, the Si|*meso*TiO_2 electrodes were UV-ozone treated for 10 min in a UV/Ozone ProCleanerTM (BioForce Nanosciences). One aliquot of H_2ase solution (1 µL, 8 pmol) was drop-cast onto each electrode and allowed to fully dry, yielding the final Si|*meso*TiO_2|H_2ase electrode.

All photocathodes were subsequently back-contacted and insulated by an epoxy adhesive prior to further use. Sand paper was used to abrade the surface of the electrode's unpolished side before application of a conductive silver paint (RS® Components 186-3593), after which an electrical wire was connected to the dry silver using the same conductive silver paint. Upon drying, a dark grey epoxy adhesive (Loctite® Hysol® 3423) was applied on both sides of the electrodes, leaving only the

surface to be analyzed (S ≈ 0.2 cm^2) exposed. The cells were then allowed to dry thoroughly for 18 h in air before use.

Characterisation of Photoelectrodes

SEM images were recorded on a FEI Philips XL30 FEG ESEM instrument at 5 kV acceleration voltage. ATR FT-IR spectra of the compounds or the functionalised TiO_2 were recorded on a Nicolet iS50 spectrometer. XPS was performed on an ESCALAB 250Xi spectrometer (Thermo Fisher Scientific, East Grindstead, UK) utilising a monochromatic Al-Kα source (50-300 W, 0.2-1 mm spot size). The quantification of the amount of immobilised **NiP** or **CoP**[3] (mole per geometrical area) on the Si|*meso*TiO_2|catalyst electrodes was evaluated by UV-visible spectroscopy after desorption of the catalyst from the corresponding electrode. Typically, the Si|*meso*TiO_2|catalyst electrode (S ≈ 0.5 cm^2) was immersed for 1.5 h in a MeOH bath containing tetrabutylammonium hydroxide (0.1 M). The **NiP** and **CoP**[3] solutions' absorptions were then measured (l = 1 cm) at 350 and 400 nm, respectively, and the concentration was estimated using the molar absorption curves in Figure S7. UV-vis spectra were collected using a Varian Cary 50 Bio UV-vis spectrometer.

Photoelectrochemical Studies

LSVs and CPP were performed with an Ivium CompactStat potentiostat. A Newport Oriel Xenon 150 W solar light simulator (100 mW cm^{-2}, AM1.5G and IR water filters, λ > 400 nm) was used as the light source. A three-electrode configuration was employed in a custom-made airtight two-compartment PEC cell with a Nafion membrane separating the compartments. A platinum mesh was used as counter electrode and an Ag/AgCl/$KCl_{(sat.)}$ electrode as reference electrode. All electrochemical measurements were performed at room temperature in aqueous acetic acid solutions (0.1 M, pH 3.0 or 4.5), except for Si|*meso*TiO_2|H_2ase and related control experiments, where a MES buffer (2-(N-morpholino)ethanesulfonic acid, 50 mM, pH 6.0) was used. LSVs were conducted at a scan rate of 5 mV s^{-1} with chopped light alternating between dark and light every 5 s. The onset potential was defined as the potential at which a photocurrent density of $|J|$ = 10 μA cm^{-2} was achieved by the respective electrode. The applied potential during CPP was 0.0 V *vs.* RHE, and continuous illumination was maintained, apart from hourly dark chops lasting for 2 min each. CPP of **CoP**[3] and H_2ase was ceased after 4 h and 5 h, respectively; all others were continued for 24 h.

Prior to the CPP experiments, the electrolyte solution in both compartments of the PEC cell was purged with N_2 containing 2 % CH_4 as an internal standard for gas chromatography (GC) measurements. The amount of gaseous H_2 was analyzed by headspace gas analysis using an Agilent 7890A Series GC equipped with a 5 Å molecular sieve column (N_2 carrier gas at a flow rate of approximately 3 mL min^{-1}). The GC oven holding the columns was kept isothermal at 45 °C, and a thermal conductivity detector was employed. Aliquots (75 μL) of the headspace gas were removed for GC analysis at regular time intervals. The FE of the photocathodes was calculated by comparing the expected amount of H_2 produced as indicated by the total charge passed through the electrode and the actual amount produced. Analytical measurements were performed in triplicate and the standard deviation of each data point is denoted by error bars.

IPCE Measurements

IPCE measurements were conducted in the same electrochemical cell set-up as used for PEC performance experiments, with the solar light simulator coupled to a monochromator (MSH300, LOT Quantum design). The sequence carried out at each wavelength was 1 min of illumination, followed by 5 min in the dark. The current was collected at two points per second, with the initial 10 and final 10 points of each light cycle averaged; the electrode's dark current was subtracted from this average to give the final photocurrent. Sample photocurrent data were normalised to the output of a power meter (Thorlabs PM100D Compact Power and Energy Meter Console). Measurements were performed in triplicate and the standard deviation at each wavelength is denoted by error bars.

Analysis of TiO_2 Charging Current

The charging and discharging of TiO_2's CB were studied by two successive chronoamperometric experiments, conducted on Si|*meso*TiO_2 and Si|*meso*TiO_2|**NiP** electrodes each. These were conducted at room temperature in a one-compartment PEC cell in a three-electrode configuration with an acetic acid solution (0.1 M, pH 4.5). In the first chronoamperometry phase, a potential of 0.0 V *vs.* RHE was applied for two min under solar light illumination (AM1.5G, 100 mW cm^{-2}, $\lambda > 400$ nm), corresponding to the charging of the CB of TiO_2. After this first phase, 20 sec were allowed to pass where the electrode was left in the dark with no applied potential. In the following second chronoamperometry phase, corresponding to the discharging step, 0.0 V *vs.* RHE was applied in the dark. In some cases, a solution of MV in the electrolyte solution was injected partway through the second chronoamperometry (final concentration in PEC cell = 10 mM). The recorded current is normalised and given as a percentage. A similar experiment was also conducted on Si|*meso*TiO_2|NiP (without the addition of MV).

Supporting Tables

Table S1. Previously published photocathodes with an immobilised molecular catalyst for proton reduction in water. All potentials are reported against RHE.

Supporting photoelectrode	Molecular co-catalyst	pH	J @ E_{app} [a] (mA cm^{-2} / V)	E_{onset} [a] (V)	FE @ E_{app} [b] (/ V)	Reported stability @ E_{app} [b] (% loss / V)	Reported photoelectrolysis duration @ E_{app} [b] (min / V)	TON (t)	Electrolyte	Illumination Power	Ref.
GaP	Cobaloxime	7.0	≈ 2.70 @ 0.0	0.76	n/a	17 @ 0.17 (after 5 min)	5 @ 0.17	n/a	1 M phosphate	100 mW cm^{-2}	4
		4.5	≈ 1.10 @ 0.0	[0.5-0.6]	0.97 @ − 0.12	18 @ − 0.12 (after 15 min)	15 @ −0.12	n/a	0.1 M acetate	100 mW cm^{-2}	5
		7.0	0.92 @ 0.0	0.72	0.88@ 0.0	n/a	30 @ 0.17	n/a	0.1 M phosphate	100 mW cm^{-2}	6
		7.0	1.3 @ 0.0	0.61	≈ 1.0 @ 0.0	≈ 27 @ 0.0 (after 60 min)	60 @ 0.0	n/a	0.1 M phosphate	100 mW cm^{-2}	7
		7.0	0.89 @ 0.0	0.65	≈ 1.0 @ 0.0	13 @ 0.0 (after 55 min)	60 @ 0.0	n/a	0.1 M phosphate	100 mW cm^{-2}	8
GaP	Cobalt-porphyrin	7.0	1.30 @ 0.0	≈ 0.55	≈ 0.97 @ 0.0	negligible loss @ 0.0 (after 4 h)	240 @ 0.0	n/a	0.1 M phosphate	100 mW cm^{-2}	9
p-GaInP$_2$ | TiO$_2$ | catalyst | TiO$_2$	Cobaloxime	13	9.00 @ 0.0	0.70	≈ 1.0 @ 0.0	≈ 5 @ 0.0 (after 20 min)	1200 @ 0.0	1.4×10^{5} (20 h)	NaOH$_{aq}$	100 mW cm^{-2}	10
InP	**[Fe$_2$S$_2$(CO)$_6$]**	7.0	≈ 0.045×10^{6} @ 0.0	0.51	≈ 0.60 @ 0.21	n/a	60 @ 0.21	n/a	0.1 M NaBF$_4$	n/a	11
P3HT:PCBM	Cobaloxime	4.5	≈ 0.002 @ 0.0	n/a	n/a	n/a	1.3 @ 0.17	n/a	0.1 M acetate	100 mW cm^{-2}	12
NiO | Al$_x$O$_y$ | **Ru(bpy)$_3$**	Cobaloxime	7.0	≈ 0.020 @ 0.2	≈ 0.87	≈ 0.68 @ 0.51	negligible loss @ 0.51 (after 1.5 h)	150 @ 0.51	n/a	0.1 M KH$_2$PO$_4$ & 0.4 M Na$_2$CO$_3$	300 W lamp	13
NiO | **PMI-6T-TPA**	**PMI-6T-TPA**	7.0	≈ 0.0020 @ 0.6	n/a	≈ 1.00 @ 0.61	negligible loss @ 0.61 (after 4 h)	240 @ 0.61	n/a	0.1 M Na$_2$SO$_4$	300 W lamp	14
NiO | **P1**	Cobaloxime	7.0	≈ 0.044 @ 0.0	n/a	0.68 @ 0.41	43 @ 0.41 (after 1.5 h)	10 @ 0.41	n/a	0.05 M phosphate	100 mW cm^{-2}	15
NiO | **RuP**	Cobaloxime	7.0	0.013 @ 0.2[b]	n/a	n/a	n/a	2.75 @ 0.20	n/a	0.07 M phosphate	300 mW cm^{-2}	16

ITO | **RuP$_2$**	Ni-DuBois (**NiP**)	5.1	0.056 @ 0.05 [b]	n/a	0.53 @ 0.05	59 @ 0.05 (after 4 h)	240 @ 0.05	n/a	0.1 M MES	445 nm 5 mW cm^{-2}	17
NiO | **RuP$_3$**	**NiP**	3.0	≈ 0.0060 @ 0.0	n/a	0.1@ 0.30	≈ 50 @ 0.30 (after 3 h)	180 @ 0.30	n/a	0.05 M Na_2SO_4	100 mW cm^{-2}	18
NiO | CdSe (QD[c])	Cobaloxime	6.8	≈ 0.110 @ 0.2 [b]	n/a	0.81@ 0.07	17 @ 0.07 (after 3.5 h)	210 @ 0.07	n/a	0.1 M Na_2SO_4	300 W lamp	19
NiO | **coumarin 343**	**$Fe_2(CO)_6$(bdt)**	4.5	0.010 @ 0.16 [b]	n/a	≈ 0.50 @ 0.16	n/a	18 @ 0.16	n/a	acetate (molarity not given)	LED lamp, cool white (5000K)	20
p-Si | *meso*TiO_2	**NiP**	4.5	0.340 @ 0.0	≈ 0.40	≈ 0.8 @ 0.0	50 @ 0.0 (after 8 h)	1440 @ 0.0	646 (24 h)	0.1 M acetate	100 mW cm^{-2}	This work

Extracted from: [a]LSV measurements, unless otherwise stated; [b]chronoamperometry measurements; [c]QD: quantum dot

Supporting References

(1) Gross, M. A.; Reynal, A.; Durrant, J. R.; Reisner, E. *J. Am. Chem. Soc.* **2014**, *136*, 356-366.
(2) Willkomm, J.; Muresan, N. M.; Reisner, E. *Chem. Sci.* **2015**, *6*, 2727-2736.
(3) Hatchikian, E. C.; Bruschi, M.; Le Gall, J. *Biochem. Biophys. Res. Commun.* **1978**, *82*, 451-461.
(4) Krawicz, A.; Yang, J.; Anzenberg, E.; Yano, J.; Sharp, I. D.; Moore, G. F. *J. Am. Chem. Soc.* **2013**, *135*, 11861-11868.
(5) Cedeno, D.; Krawicz, A.; Doak, P.; Yu, M.; Neaton, J. B.; Moore, G. F. *J. Phys. Chem. Lett.* **2014**, *5*, 3222-3226.
(6) Krawicz, A.; Cedeno, D.; Moore, G. F. *Phys. Chem. Chem. Phys.* **2014**, *16*, 15818-15824.
(7) Beiler, A. M.; Khusnutdinova, D.; Jacob, S. I.; Moore, G. F. *Ind. Eng. Chem. Res.* **2016**, *55*, 5306-5314.
(8) Beiler, A. M.; Khusnutdinova, D.; Jacob, S. I.; Moore, G. F. *ACS Appl. Mater. Interfaces* **2016**, *8*, 10038-10047.
(9) Khusnutdinova, D.; Beiler, A. M.; Wadsworth, B. L.; Jacob, S. I.; Moore, G. F. *Chem. Sci.* **2017**, *8*, 253-259.
(10) Gu, J.; Yan, Y.; Young, J. L.; Steirer, K. X.; Neale, N. R.; Turner, J. A. *Nat. Mater.* **2016**, *15*, 456-460.
(11) Nann, T.; Ibrahim, S. K.; Woi, P.-M.; Xu, S.; Ziegler, J.; Pickett, C. J. *Angew. Chem. Int. Ed.* **2010**, *49*, 1574-1577.
(12) Chen, Y.; Chen, H.; Tian, H. *Chem. Commun.* **2015**, *51*, 11508-11511.
(13) Ji, Z.; He, M.; Huang, Z.; Ozkan, U.; Wu, Y. *J. Am. Chem. Soc.* **2013**, *135*, 11696-11699.
(14) Tong, L.; Iwase, A.; Nattestad, A.; Bach, U.; Weidelener, M.; Gotz, G.; Mishra, A.; Bauerle, P.; Amal, R.; Wallace, G. G.; Mozer, A. J. *Energy Environ. Sci.* **2012**, *5*, 9472-9475.
(15) Li, F.; Fan, K.; Xu, B.; Gabrielsson, E.; Daniel, Q.; Li, L.; Sun, L. *J. Am. Chem. Soc.* **2015**, *137*, 9153-9159.
(16) Fan, K.; Li, F.; Wang, L.; Daniel, Q.; Gabrielsson, E.; Sun, L. *Phys. Chem. Chem. Phys.* **2014**, *16*, 25234-25240.
(17) Shan, B.; Das, A. K.; Marquard, S.; Farnum, B. H.; Wang, D.; Bullock, R. M.; Meyer, T. J. *Energy Environ. Sci.* **2016**, *9*, 3693-3697.
(18) Gross, M. A.; Creissen, C. E.; Orchard, K. L.; Reisner, E. *Chem. Sci.* **2016**, *7*, 5537-5546.
(19) Meng, P.; Wang, M.; Yang, Y.; Zhang, S.; Sun, L. *J. Mater. Chem. A* **2015**, *3*, 18852-18859.
(20) Antila, L. J.; Ghamgosar, P.; Maji, S.; Tian, H.; Ott, S.; Hammarström, L. *ACS Energy Letters* **2016**, *1*, 1106-1111.

Table S2. Quantification of molecular catalysts loaded on the surface of the different electrodes as estimated by UV-Vis spectroscopy measurements. Loading is given per geometrical surface area.

**Architecture**	**Thickness of the TiO_2 layer (μm)**	**Amount of immobilised catalyst (nmol cm^{-2})**
Si|*meso*TiO_2|**CoP**^{3}	6.0	93.9 ± 8.9
Si|*meso*TiO_2|**NiP**	1.1	5.6 ± 1.4
Si|*meso*TiO_2|**NiP**	6.0	38.3 ± 4.2

Supporting Figures

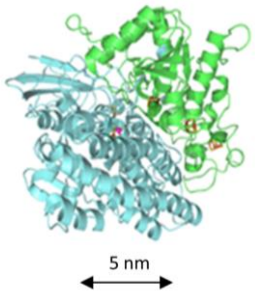

Figure S1. Structure of [NiFeSe]-hydrogenase from Garcin, E. *et al. Structure* **1999**, *7*, 557-566.

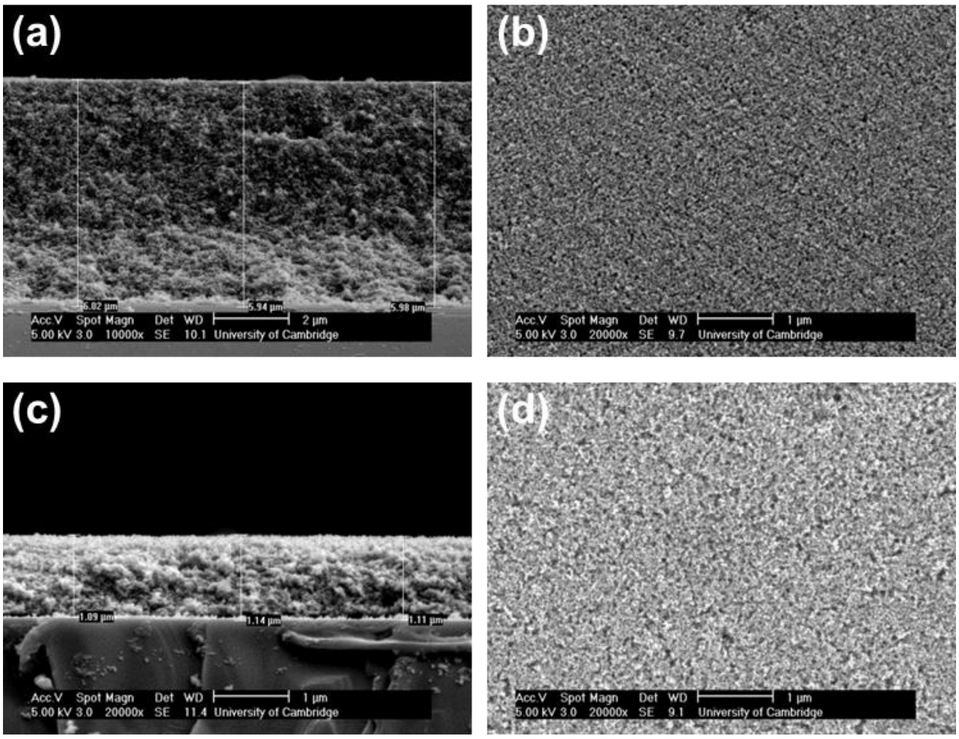

Figure S2. SEM images of Si|*meso*TiO_2 electrodes with (a-b) *meso*TiO_2 thickness = 6 µm and (c-d) *meso*TiO_2 thickness = 1 µm, as viewed in cross-section and from the top, respectively.

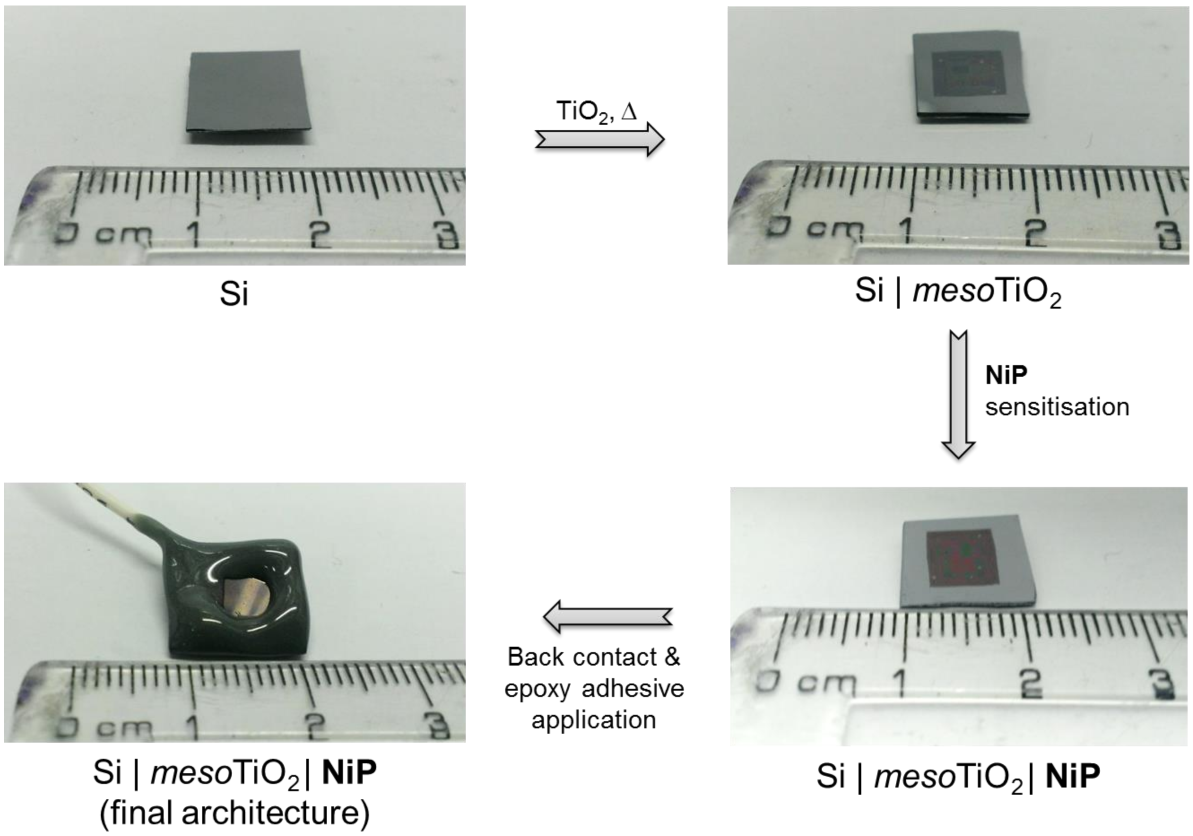

Figure S3. Photographs of typical electrodes at various stages of preparation towards the final Si|*meso*TiO_2|**NiP** photocathode.

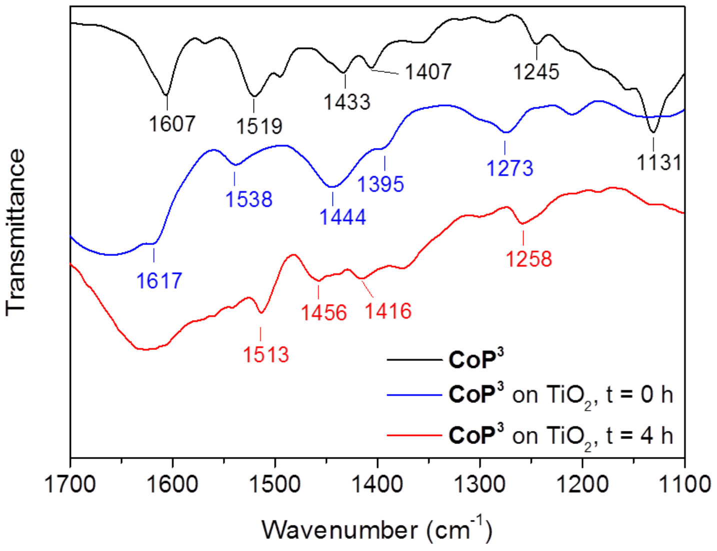

Figure S4. ATR-FTIR spectra of **CoP**^{3} (black), **CoP**^{3} on TiO_2 before CPP (blue) and **CoP**^{3} on TiO_2 after 4 h of CPP on Si|*meso*TiO_2|**CoP**^{3} electrode (red).

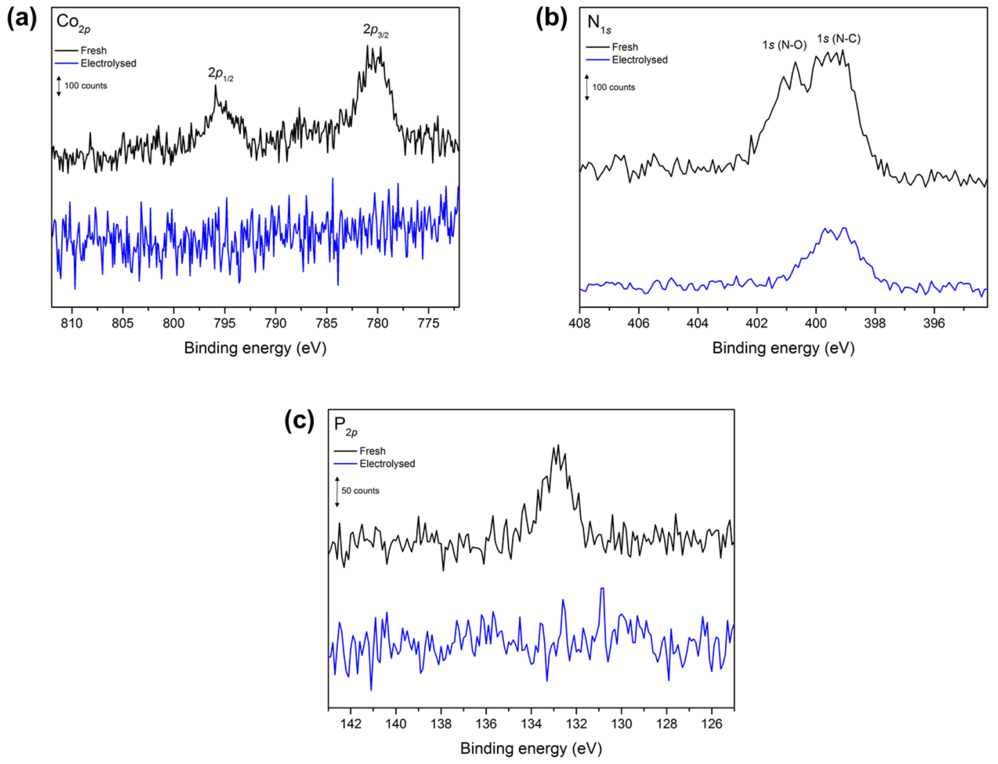

Figure S5. XPS analysis of Si|*meso*TiO_2|**CoP**[3] electrodes in the (a) Co_{2p}, (b) N_{1s} and (c) P_{2p} regions, fresh (black traces) and after 30 minutes of CPP (blue traces).

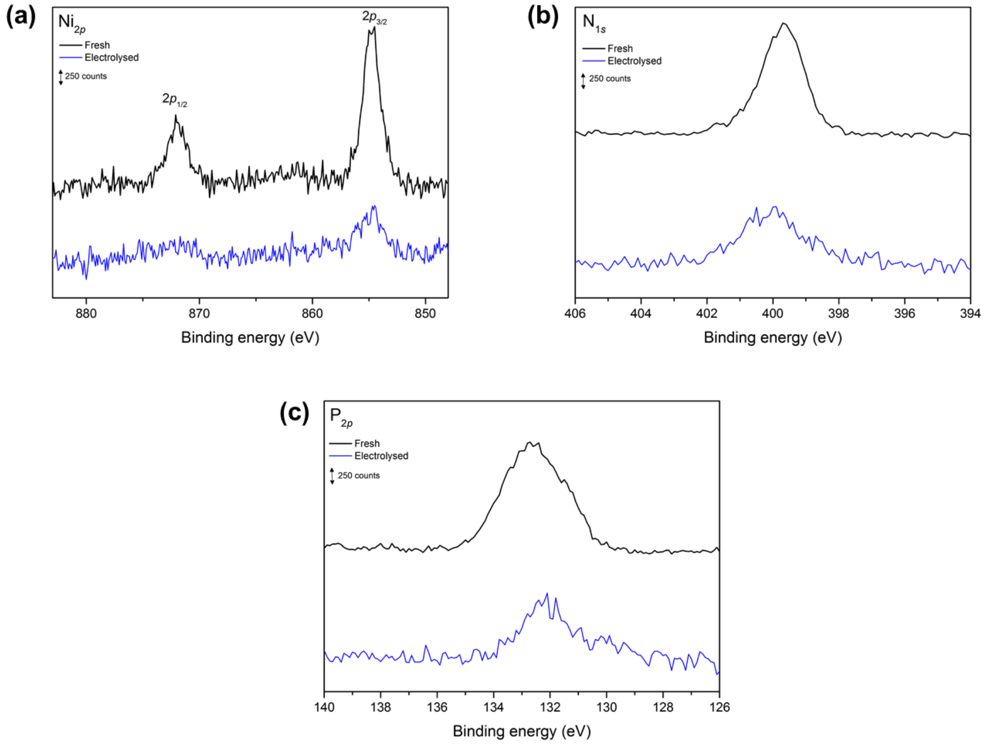

Figure S6. XPS analysis of Si|*meso*TiO_2|**NiP** electrodes in the (a) Ni_{2p}, (b) N_{1s} and (c) P_{2p} regions, fresh (black traces) and after 1 h of CPP (blue traces).

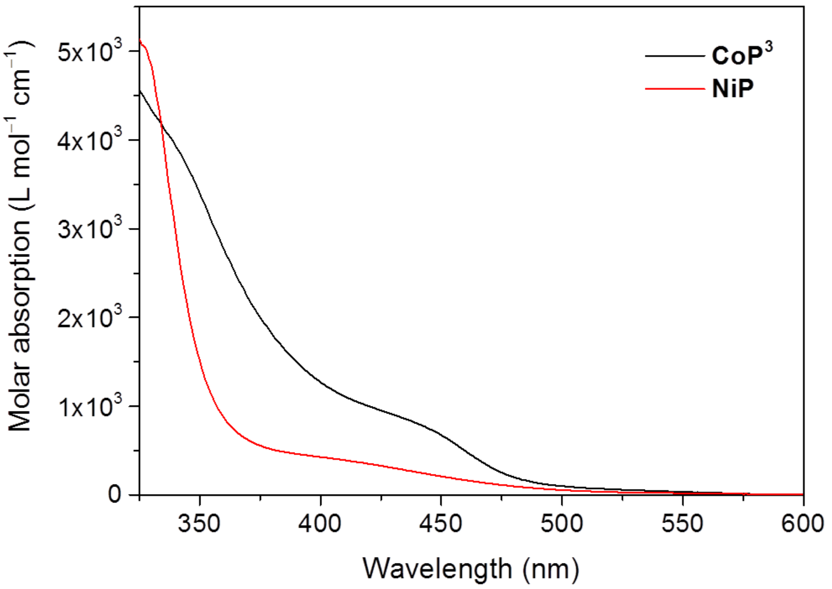

Figure S7. UV-visible spectra of **CoP**[3] (black) & **NiP** (red) solubilised in methanol in the presence of tetrabutylammonium hydroxide (0.1 M), measured at room temperature.

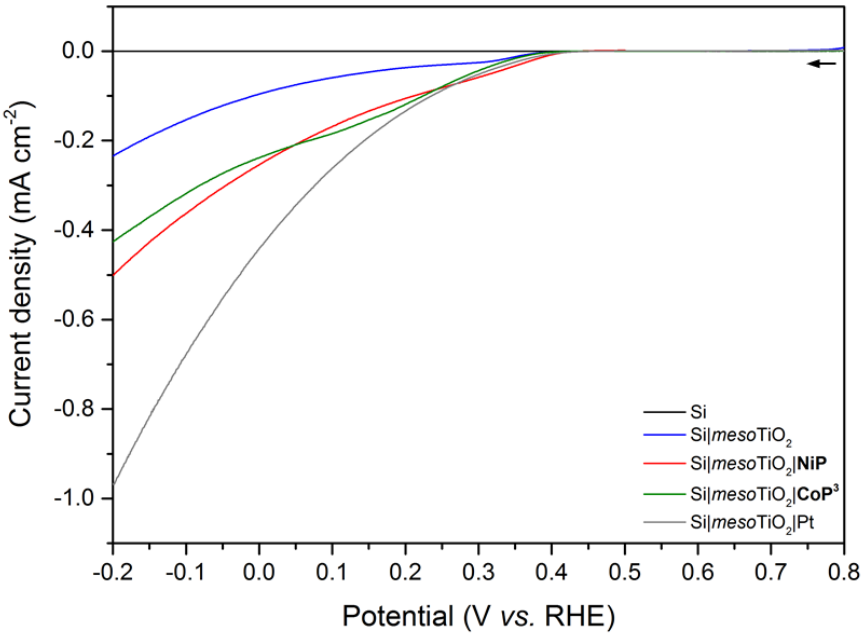

Figure S8. LSVs under constant UV-filtered simulated solar illumination (AM1.5G, 100 mW cm^{-2}, $\lambda > 400$ nm) of Si, Si|*meso*TiO_2, molecular catalyst-sensitised Si|*meso*TiO_2|**NiP** and Si|*meso*TiO_2|**CoP**[3], and Pt-loaded Si|*meso*TiO_2|Pt electrodes. Conditions: aqueous acetic acid buffer (0.1 M, pH 4.5, no catalyst in solution), N_2 atmosphere, room temperature; scan rate $\upsilon = 5$ mV s^{-1}.

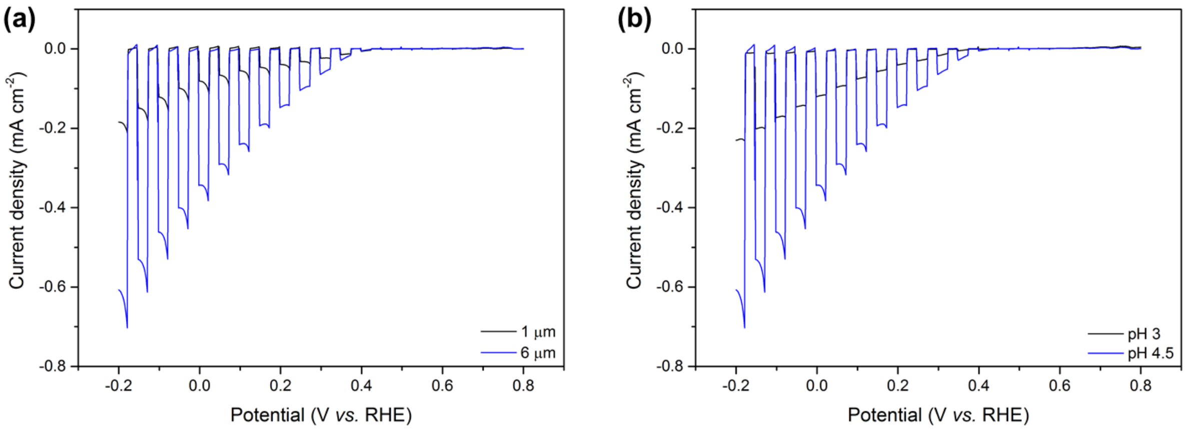

Figure S9. LSVs under chopped UV-filtered simulated solar (AM1.5G, 100 mW cm^{-2}, λ > 400 nm) of Si|*meso*TiO_2|**NiP** electrodes, (a) prepared with two different thicknesses of the *meso*TiO_2 layer and tested under pH 4.5 conditions, and (b) prepared with a *meso*TiO_2 thickness of 6 µm and tested under two different pH conditions. Conditions: aqueous acetic acid buffer (0.1 M, pH 3 or 4.5, no catalyst in solution), N_2 atmosphere, room temperature scan rate υ = 5 mV s^{-1}.

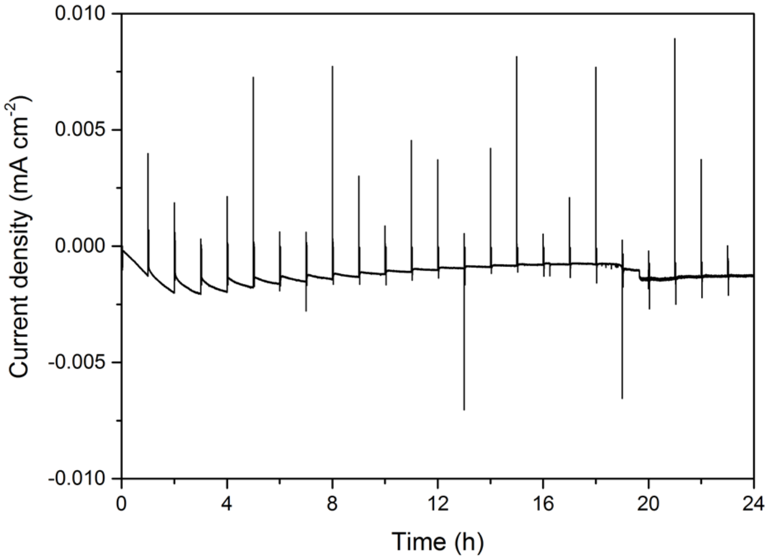

Figure S10. Chronoamperogram from CPP held at 0.0 V *vs.* RHE over a 24 hour period of bare Si. Conditions: aqueous acetic acid buffer (0.1 M, pH 4.5), N_2 atmosphere with internal CH_4 standard, room temperature, constant illumination (AM1.5G, 100 mW cm^{-2}, λ > 400 nm) with hourly dark chop.

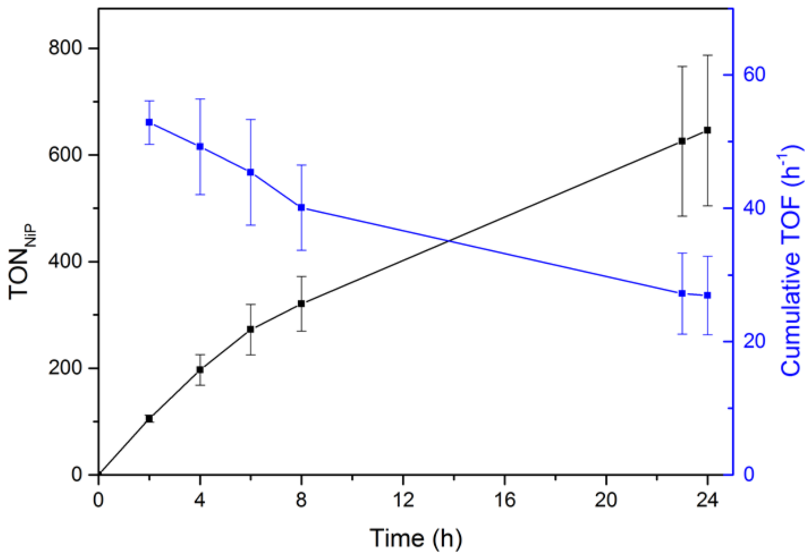

Figure S11. Cumulative turnover number and turnover frequency of **NiP** over 24-hour CPP of Si|*meso*TiO_2|**NiP** (background H_2 production by a Si|*meso*TiO_2 control electrode has been subtracted). Conditions: aqueous acetic acid buffer (0.1 M, pH 4.5), N_2 atmosphere with internal CH_4 standard, room temperature, constant illumination (AM1.5G, 100 mW cm^{-2}, $\lambda > 400$ nm) with hourly dark chop.

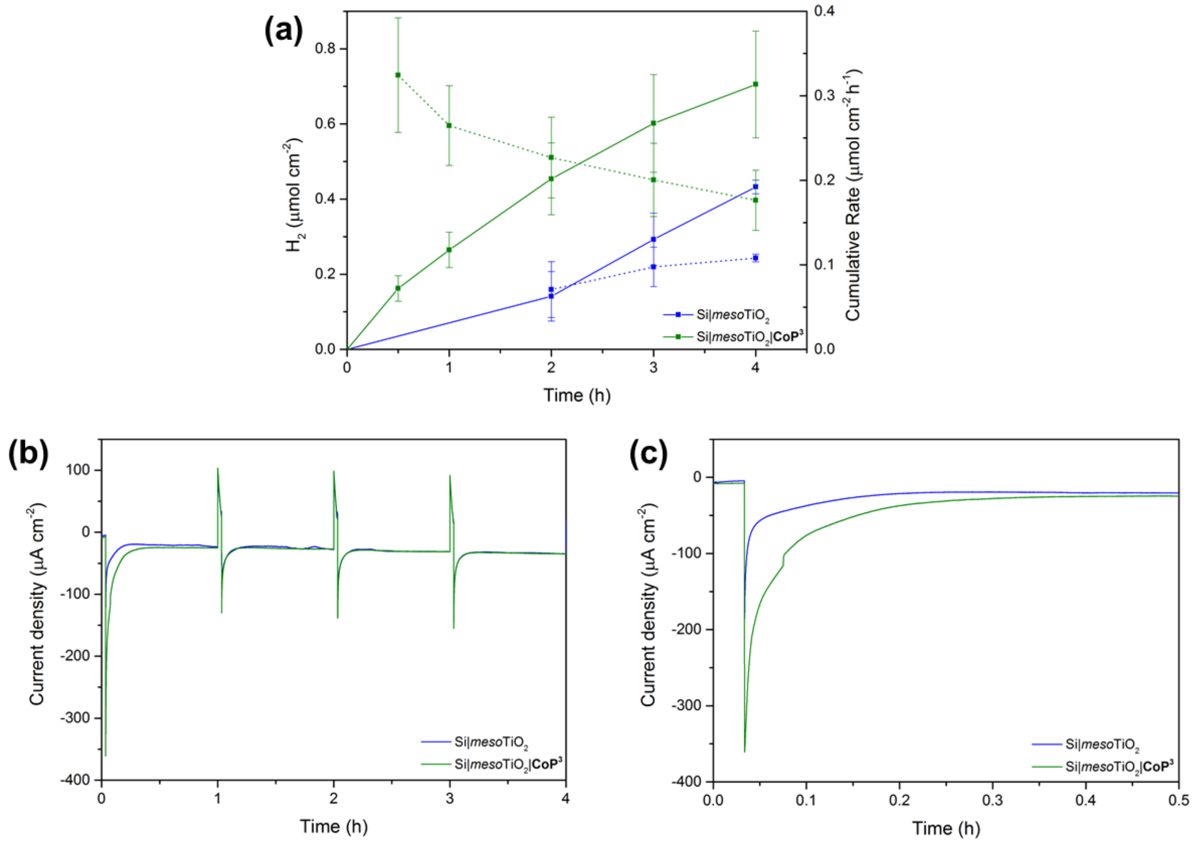

Figure S12. CPP data for chronoamperometry performed for 4 hours under constant UV-filtered simulated solar illumination (AM1.5G, 100 mW cm^{-2}, $\lambda > 400$ nm) with an hourly dark chop lasting for two minutes each, held at 0.0 V *vs.* RHE for Si|*meso*TiO_2 and Si|*meso*TiO_2|**CoP**[3]: (a) H_2 evolution (solid lines) and cumulative turnover frequency (dashed lines); (b) chronoamperograms over a 4 hour period and (c) with a close-up view of the first 30 minutes. Conditions: aqueous acetic acid buffer (0.1 M, pH 4.5), N_2 atmosphere with internal CH_4 standard, room temperature.

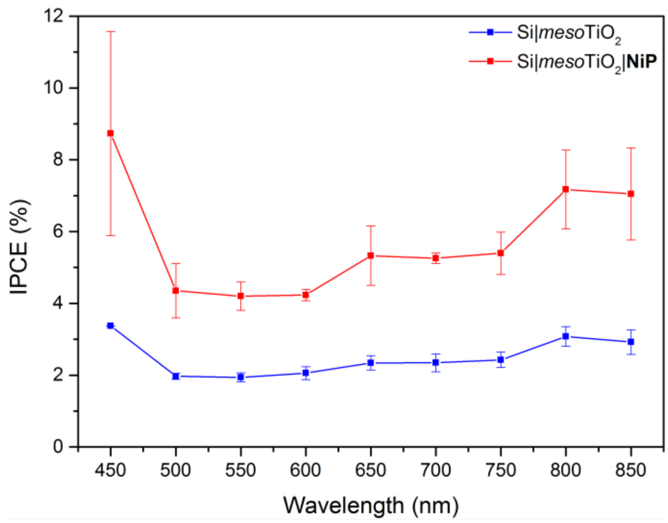

Figure S13. IPCE spectra of Si|*meso*TiO_2 and Si|*meso*TiO_2|NiP, measured at E_{app} = 0.0 V *vs.* RHE. Conditions: aqueous acetic acid buffer (0.1 M, pH 4.5), N_2 atmosphere, room temperature.

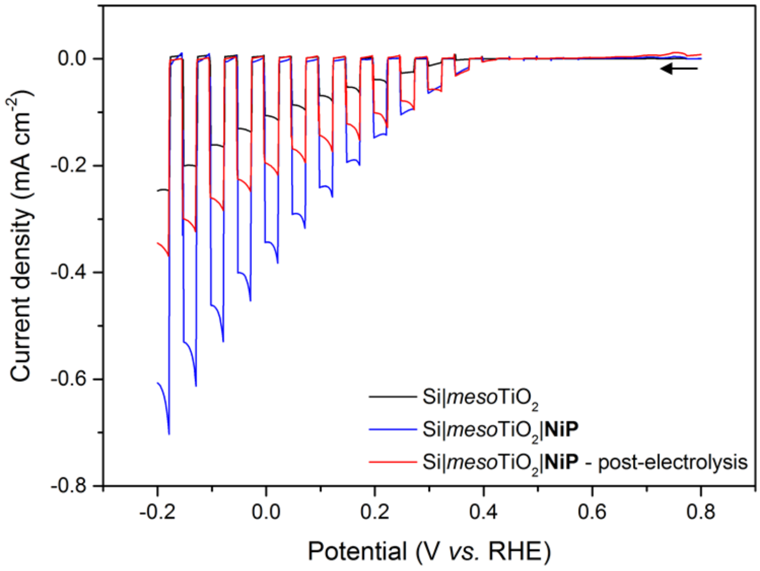

Figure S14. LSVs of. Si|*meso*TiO_2, fresh Si|*meso*TiO_2|**NiP** and electrolysed Si|*meso*TiO_2|**NiP** photocathodes (after 24 h CPP). Conditions: aqueous acetic acid buffer (0.1 M, 4.5), N_2 atmosphere, room temperature; scan rate υ = 5 mV s^{-1}.

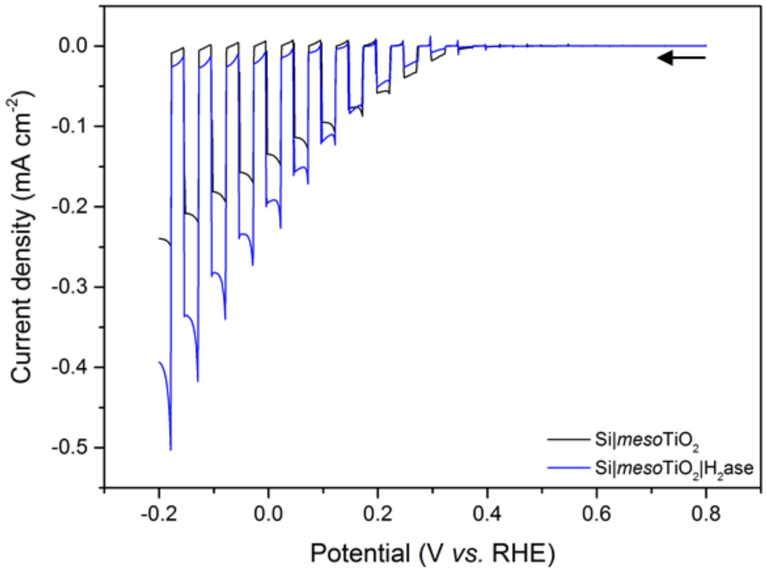

Figure S15. LSVs under chopped illumination (AM1.5G, 100 mW cm^{-2}, λ > 400 nm) of Si|*meso*TiO_2 and Si|*meso*TiO_2|H_2ase. Conditions: aqueous MES buffer (50 mM, pH 6.0), N_2 atmosphere, room temperature; scan rate υ = 5 mV s^{-1}.

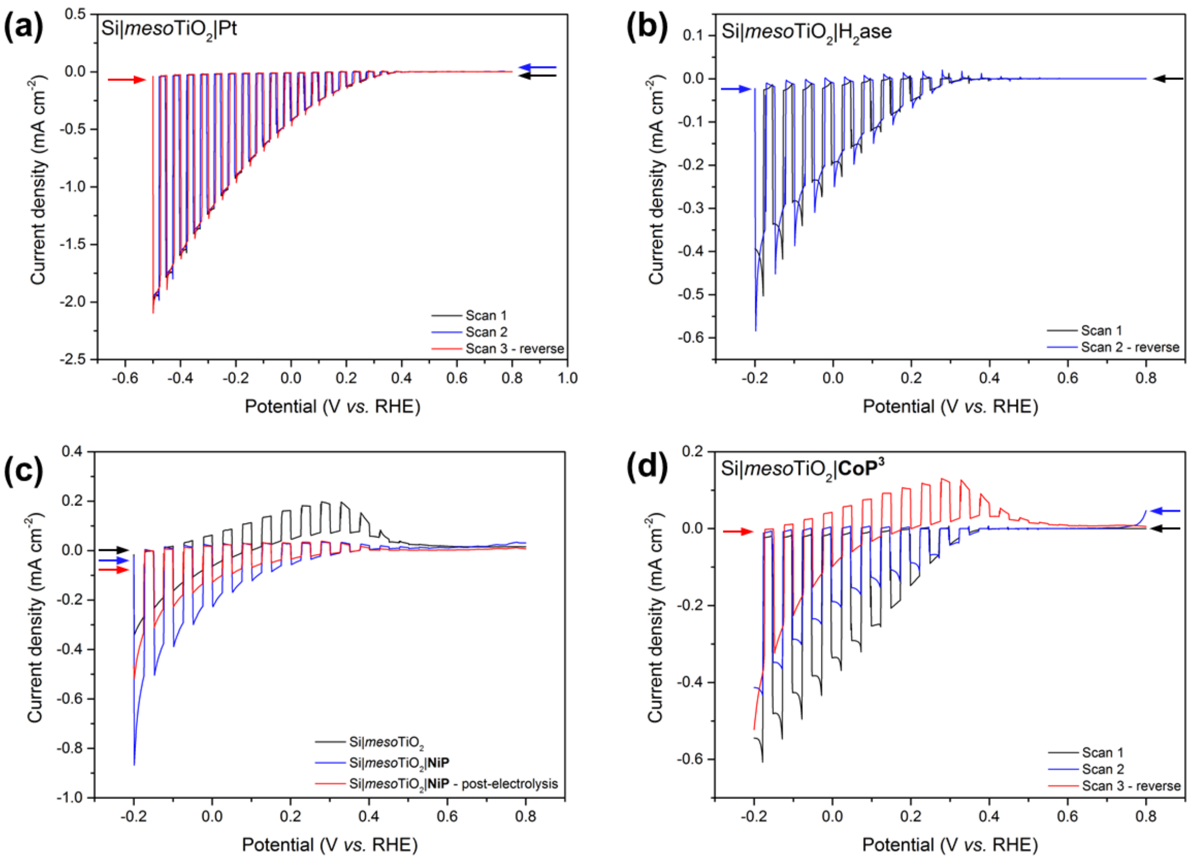

Figure S16. LSVs under chopped illumination (AM1.5G, 100 mW cm^{-2}, λ > 400 nm) of (a) Si|*meso*TiO_2|Pt, (b) Si|*meso*TiO_2|H_2ase and (d) Si|*meso*TiO_2|**CoP^3** electrodes, first scanning once/twice in the direction of increasingly reducing potentials and then once in the opposite direction, and (c) of Si|*meso*TiO_2, fresh Si|*meso*TiO_2|**NiP** and electrolysed Si|*meso*TiO_2|**NiP** photocathodes, scanning in the direction of increasing oxidising potentials. Arrows indicate scanning direction in all LSVs. Conditions: aqueous acetic acid buffer (0.1 M, pH 4.5) for Si|*meso*TiO_2|Pt and Si|*meso*TiO_2|**CoP^3**, aqueous MES buffer (50 mM, pH 6) for Si|*meso*TiO_2|H_2ase; N_2 atmosphere, room temperature; scan rate υ = 5 mV s^{-1}.

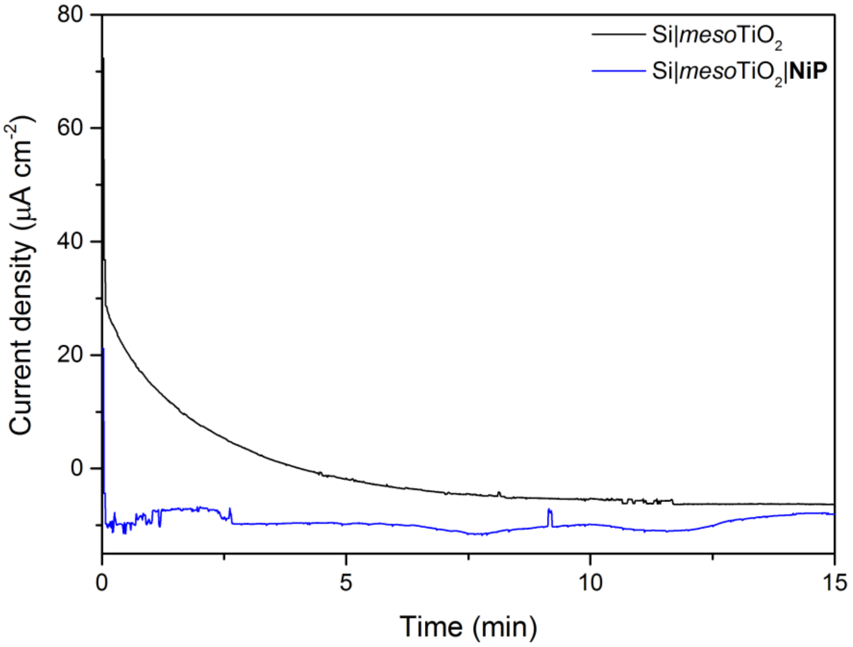

Figure S17. Chronoamperograms of Si|*meso*TiO$_2$ and Si|*meso*TiO$_2$|**NiP** electrodes in the dark after having first been exposed to light; $E_{applied}$ during both light and dark phases = 0.0 V *vs.* RHE. Conditions: aqueous acetic acid buffer (0.1 M, pH 4.5), N_2 atmosphere, room temperature.

End of Supporting Information
